# *Lagoa Misteriosa*, a novel source of beetle diversity: two new species of Brazilian riffle beetles *(Insecta: Coleoptera: Elmidae: Elminae)* with immatures from a karst sinkhole

**DOI:** 10.1007/s13744-026-01441-3

**Published:** 2026-10-23

**Authors:** Thiago Tadeu Silva Polizei, Éverton G. M. Neves, Sônia A. Casari, Anderson Ferreira

**Affiliations:** 1 Lab de Coleoptera (LaC), Museu de Zoologia da Universidade de São Paulo (MZSP), Av. Nazaré 481, São Paulo, SP CEP 04263-000 Brazil; 2 Instituto Tecnológico Vale (ITV), Rua Boaventura da Silva, Belém, Pará 955, CEP 66055-090 Brazil; 3 Programa de Pós-Graduação em Ecologia de Ambientes Aquáticos Continentais (PEA), Universidade Estadual de Maringá (UEM), Maringá, Paraná, Brazil; 4 Centro de Ciências Biológicas, Depto de Biologia, Lab de Ecologia de Lagos Rasos e Invasões (LESLIN), Universidade Estadual de Maringá (UEM), Maringá, Paraná, Brazil; 5 Faculdade de Ciências Biológicas e Ambientais, Lab de Ecologia Aquática (LEAqua), Univ Federal da Grande Dourados (UFGD), Dourados, Mato Grosso Do Sul Brasil; 6 Universidade Federal da Grande Dourados (UFGD), Programa de Pós-Graduação em Biodiversidade E Meio Ambiente (PPGBMA), Dourados, Mato Grosso Do Sul Brasil

**Keywords:** Aquatic insects, Biodiversity, Neotropics, Taxonomy, Neotropical Lake

## Abstract

Elmidae comprises the largest aquatic family of Dryopoidea, with 1,650 species recorded worldwide. Brazil has the most diversity with 210 described species. Although this number has increased in recent years, in some Brazilian geographical regions there are knowledge gaps in the distribution of fauna, especially based on immature stages. After a yearlong aquatic insect survey conducted in a karst sinkhole located in Jardim municipality, Mato Grosso do Sul State, Brazil, we found an endemic and novel beetle fauna which included new species. Here we present a taxonomic description of two new species of Elmidae, *Microcylloepus dimorphus* **sp. nov**. and *Hexacylloepus misteriosa* **sp. nov.**, based on males, females, and immatures. These new species represent the first Brazilian Elmidae described from a lentic habitat. Also, we provide photographic images of the habitus, SEM images, and illustrations of the diagnostic structures for both species, including their immatures, and habitat notes about Lagoa Misteriosa.

## Introduction

Elmidae comprises the largest aquatic family of Dryopoidea with 1650 species recorded worldwide. The highest richness is found in the Neotropical region, which has almost 1/3 of all known elmid genera in the world (Polizei et al. 2022). Brazil has the widest diversity with 210 described species (Polizei et al. 2026). Many new taxa have been described in recent years (Polizei et al. 2022; Fernandes et al. 2023, 2025; Almeida et al. 2024; Pinedo-Garcia et al. 2024a, 2024b), especially in the northeast and middle west regions where distribution gaps exist, particularly regarding immature stages.

The New World genus *Microcylloepus* Hinton 1935 includes 29 species recorded from 16 countries (Jäch et al. 2016; Polizei 2018). The adult diagnostic characteristics are a subparallel, strongly striate, punctate body; pronotum with a sublateral carina extending from base to apex, with transverse impression on the apical third, with a broad median impression, and with two oblique, basal impressions (Polizei 2018). The larva can be recognized by the parallel, cylindrical body, with tubercles on the first eight abdominal tergites arranged in longitudinal rows (Hinton 1940a).

The genus *Hexacylloepus* Hinton 1940a, 1940b represents one of the more diverse genera of Elmidae, with 43 described species known from 22 New World countries (Jäch et al. 2016; Polizei et al. 2020). The adult can be recognized by an elongated body; pronotum with a longitudinal sublateral carina on each side and a longitudinal impression on the disc; elytra striate and punctate, with two sublateral carinae on each elytron; hypomeron covered with a belt of tomentum (Polizei et al. 2020). The larva can be recognized by the parallel, hemicylindrical body, clypeus with an inconspicuous tooth, and the last abdominal ventrite 3x longer than wide (Brown 1973).

Larvae of only one species of each of these genera have been described, *Hexacylloepus ferrugineus* (Horn 1870) by Brown (1973) and *Microcylloepus inaequalis* (Sharp 1882) by Hinton (1940a). There are illustrations of the larval dorsal habitus of *Microcylloepus pusillus* (LeConte, 1852) (as *Helmis pusilla*) (Böving and Craighead 1931), and of both genera without specific identification.

Hinton (1940a), in his Mexican monograph, presented a larval key to the Elmidae genera, characterized *Microcylloepus* and described and illustrated the mature larva of *Microcylloepus inaequalis*. Bertrand (1955) presented illustrations of larval *Microcylloepus* antenna and ornamentation of the integument and segment IX (dorsal), and of the larval habitus (dorsal) of *M. pusillus*.

Sanderson and Brown (1956) described the larva of *Hexacylloepus ferrugineus* collected in Oklahoma. They illustrated the head (dorsal) and the fourth abdominal tergite representing the integumental ornamentations. Also presented the differences between larvae of *Hexacylloepus* and *Microcylloepus*, stating that the differences are not great. In fact, Brown (1973) verified that the larva described by Sanderson and Brown (1956) was not of *Hexacylloepus* and in reality, is that one *Microcyllopeus*. He characterized the genus *Hexacylloepus* and described and illustrated the true larva of *H. ferrugineus*.

Bertrand (1965), studying the same Oklahoma material, complemented the Sanderson and Brown (1956) description of *Hexacylloepus ferrugineus* larvae and provided original figures of the pro- and mesothorax (dorsal), segments VIII-IX (dorsal), antenna, and tergite and sternite ornamentation. He also amended his 1955 synopsis with a revised key to the genera *Hexacylloepus*, *Microcylloepus* and *Heterelmis* Sharp 1882. Brown (1987) presented the life history of Elmidae, including the adults and larvae of Elminae and Larainae.

Passos et al. (2007) presented a generic key for identification of Elmidae from Rio de Janeiro State (Brazil). They illustrated the larval habitus (dorsal and ventral) of *Microcylloepus* sp. and *Hexacylloepus* sp.

Segura et al. (2011) studied the Elmidae larvae from São Paulo State (Brazil). They presented a key to generic identification, some biological data, and discussed the importance of the family. They included photos of larvae of several genera, some with explanatory captions, including of *Microcylloepus* sp. dorsal habitus, dorsal thorax and abdominal segments I-III, and *Hexacylloepus* sp. lateral habitus and dorsal abdomen.                                                                                                                                                                                      

The increasing knowledge of Neotropical Elmidae larvae is essential for improving species identification and taxonomy, in this way, the larval morphology provides valuable diagnostic characters and supports ecological, systematic and biodiversity studies of aquatic beetles. 

In this paper, we present a morphological description of two new species of two different genera of Elmidae, *Microcylloepus dimorphus* **sp. nov.** and *Hexacylloepus misteriosa* **sp. nov.**, based on males, females and immatures from a karst sinkhole located in Jardim municipally, Mato Grosso do Sul State, Brazil. For both species, we provide photographic images of the habitus, SEM images, and illustrations of the diagnostic structures including those of the immatures, and habitat notes about Lagoa Misteriosa.

## Materials and Methods

### Study Area

**Fig. 1:**
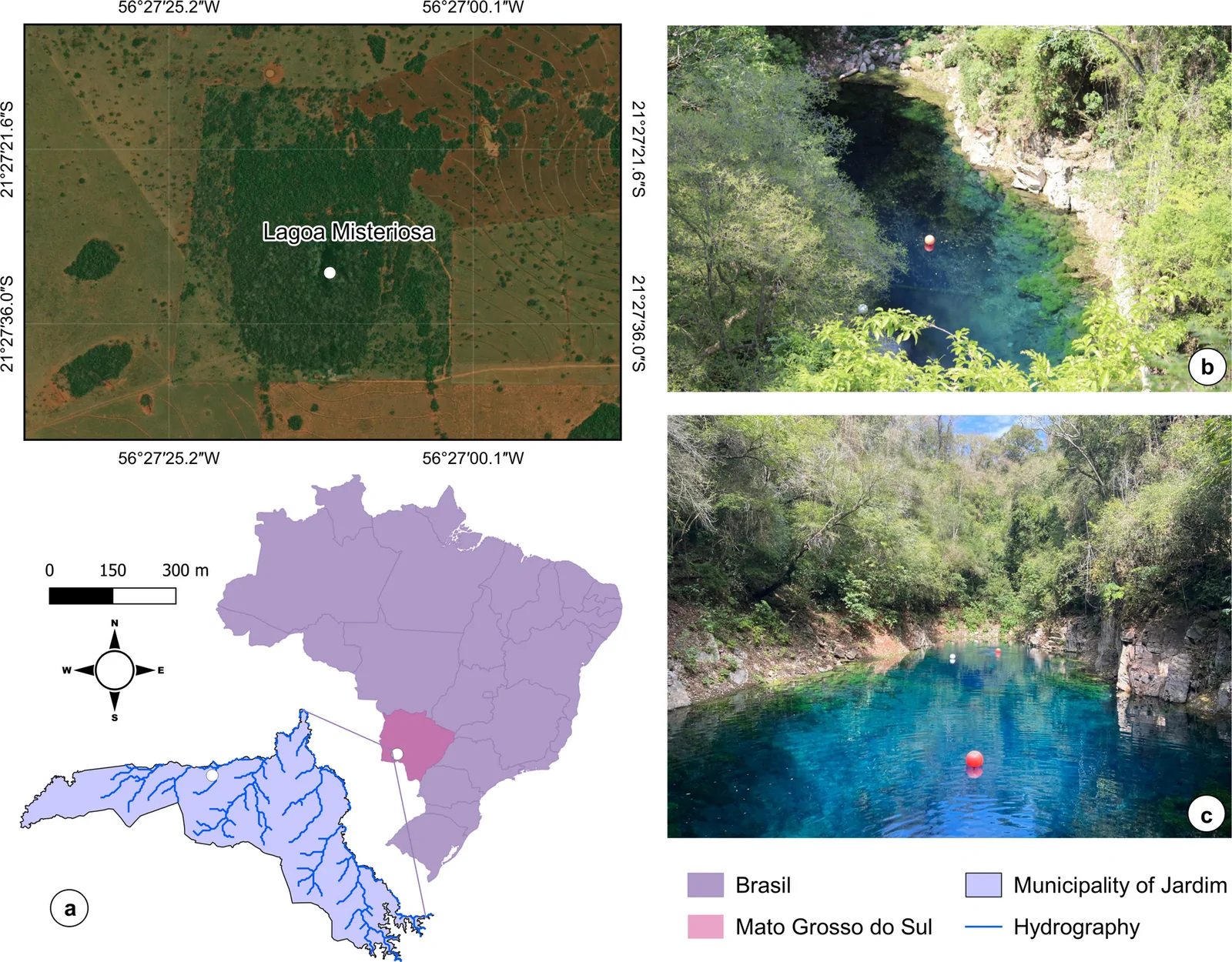
Lagoa Misteriosa. **a** Satellite view, Brazilian map, Municipality of Jardim with hydrography. **b–c** Dry season

Lagoa Misteriosa (Mysterious Lake) (21°27′32.63" S; 56°27′12.31" W) is a conservation unit located in Jardim Municipally in the southwestern region of the state of Mato Grosso do Sul State, Brazil (Fig. 1), (Scheffler 2014), within a karst sinkhole that is part of the Serra da Bodoquena karst system (Sallun-Filho et al. 2010). Lagoa Misteriosa is known as the deepest flooded cave in Brazil, featuring latero-vertical limestone walls and an underwater cavern with a depth exceeding 220 m and surface with 900 m^2^ (Sallun-Filho and Karmann 2007; Coelho 2009; Scheffler et al. 2019), however, its precise depth is still unknown. When compared with a European similar lake, it features a characteristic karst habitat, a recognized biodiversity hotspot, which generally harbors many endemic species (Simović et al. 2024).

During the hottest period of the year and the rainy season, Lagoa Misteriosa is marked annually by a phytoplankton bloom (Coelho et al. 2011; Scheffler et al. 2019), in which the water turns green. The surrounding vegetation includes phytophysiognomies of deciduous seasonal forest and wooded savanna (Coelho 2009). The average recorded minimum temperature was 13.8°C, and the maximum was 30.8°C.

### Sampling

Specimens were collected bimonthly in the Lagoa Misteriosa from October to December (2021) and February (phytoplankton bloom; visibility approximately three meters), April, June, and August (2022) (high water transparency; visibility approximately 55 m). Elmidae specimens were sampled in association with the rooted macrophyte *Heteranthera zosterifolia* (Martius 1823) (Pontederiaceae) along three vertical banks of the pond. Nine samples of the macrophyte were collected each time of collection, placed in plastic bags, labeled, and fixed in 4% formaldehyde in the field. In the laboratory, the macrophytes were washed under running water, and aquatic beetles that were on the leaves and stems were retained using granulometric sieve. Elmidae specimens were sorted and identified under a stereoscopic microscope, and individuals were preserved in 70% ethanol.

At each sampling point, measurements of hydrogen potential (pH), electrical conductivity (μS/cm), dissolved oxygen concentration (mg/L) and vertical water transparency were obtained using the HANNA HI9829 Multiparameter Analyzer (Table 1).

**Table 1:** Mean and standard deviation of the physicochemical parameters of the water in Lagoa Misteriosa

	Phytoplankton bloom			High water transparency		
	October 2021	December 2021	February 2022	April 2022	June 2022	August 2022
pH	7.55 ± 0.16	8.42 ± 0.02	8.23 ± 0.03	7.36 ± 0.16	7.19 ± 0.12	7.14 ± 0.07
Dissolved oxygen	8.22 ± 0.38	7.88 ± 0.37	7.87 ± 0.20	7.55 ± 0.16	5.95 ± 0.29	6.75 ± 0.16
Conductivity	272.89 ± 33.34	478.78 ± 1.92	233.56 ± 0.73	292.78 ± 0.44	353.00 ± 0.00	368.44 ± 0.53
Vertical Water Transparency	22.5 ± 0.01	4 ± 0.01	3 ± 0.01	31 ± 0.01	49 ± 0.01	55 ± 0.01

Larvae and adults were easily associated because only two different taxa were found during a one-year survey, and both genera are well characterized by both larvae and adults. Thus, no other Elmidae taxa were sampled throughout the entire study period.

### Morphological Studies

The morphological studies were carried out with a Leica N205A stereoscopic microscope. For the study of larval anatomy, the specimens were totally or partially cleared in cold 10% KOH. Adult mouthparts, ovipositor and male genitalia were detached from the specimens and cleared in heated 10% KOH for ten minutes. The head (larva), mouthparts (larva and adult), thorax (larva), abdominal sternites (larva), ovipositor and male genitalia were studied and illustrated using a Leica WILD M10 and Zeiss Axioskop microscope with a camera lucida.

Larvae and adults were photographed using a Canon EOS550D camera coupled to a Leica N205A stereoscopic microscope and a Leica MC120HD camera coupled to a Leica M165C stereoscopic microscope. Photographs and line drawings were edited using Adobe Photoshop CC®, Adobe Illustrator CC®.

Specimens of *Microcylloepus dimorphus* **sp. nov.** were also studied using an electron microscope. The specimens were prepared through an ascending ethanol series, remaining one day in each dilution (70%, 90% and 100%), dried through the critical point, placed on SEM specimen stubs with double-sided tape, and coated with gold. The scanning electron microscope images were taken with a Carl Zeiss LEO 440 scanning electron microscope in the Laboratório de Microscopia Eletrônica de Varredura at MZSP.

The general morphological terminology for larva and adults follows Lawrence (1991) and Kodada et al. (2016) respectively.

### Map Production

The map was produced using the freeware QGIS 3.32.0 with the biogeographical regionalization of the Neotropical region and accompanying shapefiles from National Water and Sanitation Agency (ANA) and the HydroSheds River Atlas.

### Depositories

CEMT – Coleção Entomológica Eurides Furtado, Universidade Federal do Mato Grosso. Cuiabá, Mato Grosso State, Brazil.

INPA – Coleção de Invertebrados do Instituto Nacional de Pesquisas da Amazônia. Manaus, Amazonas State, Brazil.

MZSP – Museu de Zoologia da Universidade de São Paulo. São Paulo, São Paulo State, Brazil.

## Results

*Microcylloepus dimorphus sp. nov.*

Figs. 2, 3, 4,5, 6, 7, 8, 9, 10, 11 and 12

***ZooBank Registration Number:*** https://zoobank.org/NomenclaturalActs/3243CD8D-9B01-4E72-A8F2-EA3ED6FD9E6F

***Material Examined:*** Male HOLOTYPE. ♂ BRASIL. Mato Grosso do Sul./Jardim. Lagoa Misteriosa./14.XII.2021. E. Neves et al. leg./021°27′36.0’’S 56°27′21.6’’W. (MZSP 71317). **189 PARATYPES**. 21 ♂ and 16 ♀ same data as holotype. (MZSP 71318–71354). BRASIL. Mato Grosso do Sul./Jardim. Lagoa Misteriosa./22.X.2021. E. Neves et al. leg./021°27′36.0’’S 56°27′21.6’’W. (12♂ and 14♀ MZSP 71355–71380). BRASIL. Mato Grosso do Sul./Jardim. Lagoa Misteriosa./10.II.2022. E. Neves et al. leg./021°27′36.0’’S 56°27′21.6’’W. (19♂ and 27♀ MZSP 71381–71418). BRASIL. Mato Grosso do Sul./Jardim. Lagoa Misteriosa./25.IV.2022. E. Neves et al. leg./021°27′36.0’’S 56°27′21.6’’W. (15♂ and 7♀ MZSP 71419–71440). BRASIL. Mato Grosso do Sul./Jardim. Lagoa Misteriosa./28.VI.2022. E. Neves et al. leg./021°27′36.0’’S 56°27′21.6’’W. (12♂ and 7♀ MZSP 71441–71459). BRASIL. Mato Grosso do Sul./Jardim. Lagoa Misteriosa./24.VIII.2022. E. Neves et al. leg./021°27′36.0’’S 56°27′21.6’’W. (5♂ and 5♀ in CEMT; 5♂ and 5♀ in INPA; 11♂ and 8♀ in MZSP 71460–71478). ***Additional material: Adults:*** BRASIL. Mato Grosso do Sul./Jardim. Lagoa Misteriosa./10.II.2022. E. Neves et al. leg./021°27′36.0’’S 56°27′21.6’’W. (1♂ and 1♀ under SEM, MZSP 71479–71480). **2.391 larvae**: BRASIL. Mato Grosso do Sul./Jardim. Lagoa Misteriosa./22.X.2021. E. Neves et al. leg./021°27′36.0’’S 56°27′21.6’’W. (289 larvae MZSP 10380). BRASIL. Mato Grosso do Sul./Jardim. Lagoa Misteriosa./14.XII.2021. E. Neves et al. leg./021°27′36.0’’S 56°27′21.6’’W. (635 larvae MZSP 10381). BRASIL. Mato Grosso do Sul./Jardim. Lagoa Misteriosa./10.II.2022. E. Neves et al. leg./021°27′36.0’’S 56°27′21.6’’W. (1061 larvae MZSP 10382) (6 larvae under SEM). BRASIL. Mato Grosso do Sul./Jardim. Lagoa Misteriosa./25.IV.2022. E. Neves et al. leg./021°27′36.0’’S 56°27′21.6’’W. (199 larvae MZSP 10383). BRASIL. Mato Grosso do Sul./Jardim. Lagoa Misteriosa./28.VI.2022. E. Neves et al. leg./021°27′36.0’’S 56°27′21.6’’W. (36 larvae MZSP 10384). BRASIL. Mato Grosso do Sul./Jardim. Lagoa Misteriosa./24.VIII.2022. E. Neves et al. leg./021°27′36.0’’S 56°27′21.6’’W. (171 larvae MZSP 10385).

*Diagnosis*.

**Fig. 2:**
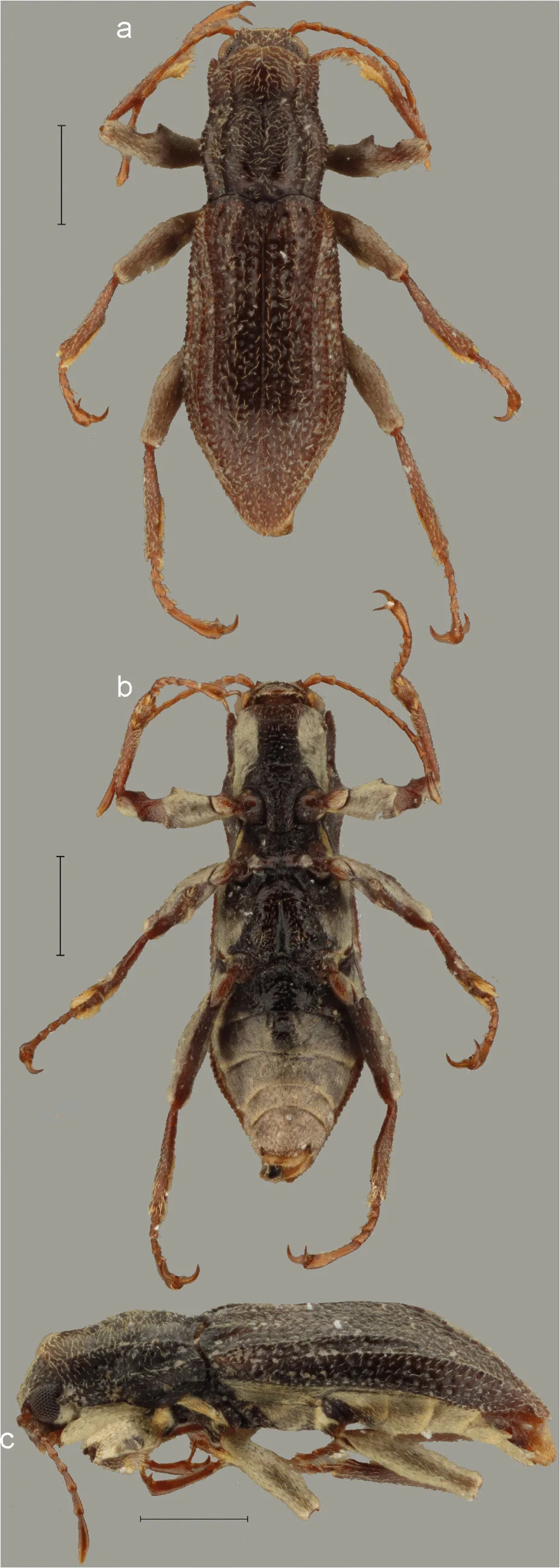
*Microcylloepus dimorphus* **sp. nov.** *Habitus*. Male. **a** Dorsal view. **b** Ventral view. **c** Lateral view. Scale bar = 0.5 mm

**Fig. 3:**
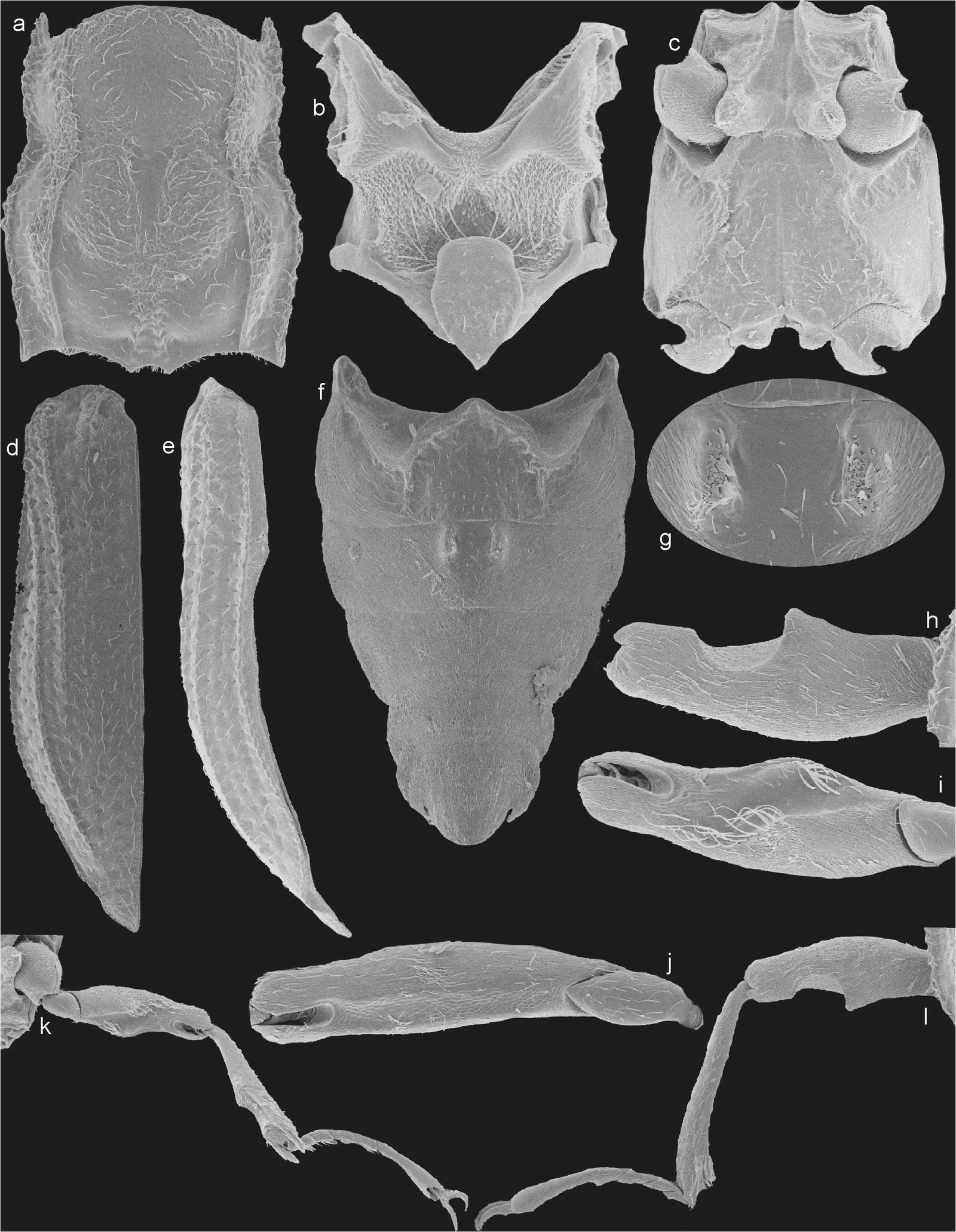
*Microcylloepus dimorphus* **sp. nov.** Male under SEM. **a** Pronotum. **b** Mesonotum. **c** Meso and metaventrite. **d** Left elytron, dorsal view. **e** Right elytron, lateral view. **f** Abdomen. **g** Disc of ventrite 2. **h** Protibiae. **i** Mesotibiae. **j** Metatibiae. **k** Mesoleg. **l** Proleg

**Fig. 4:**
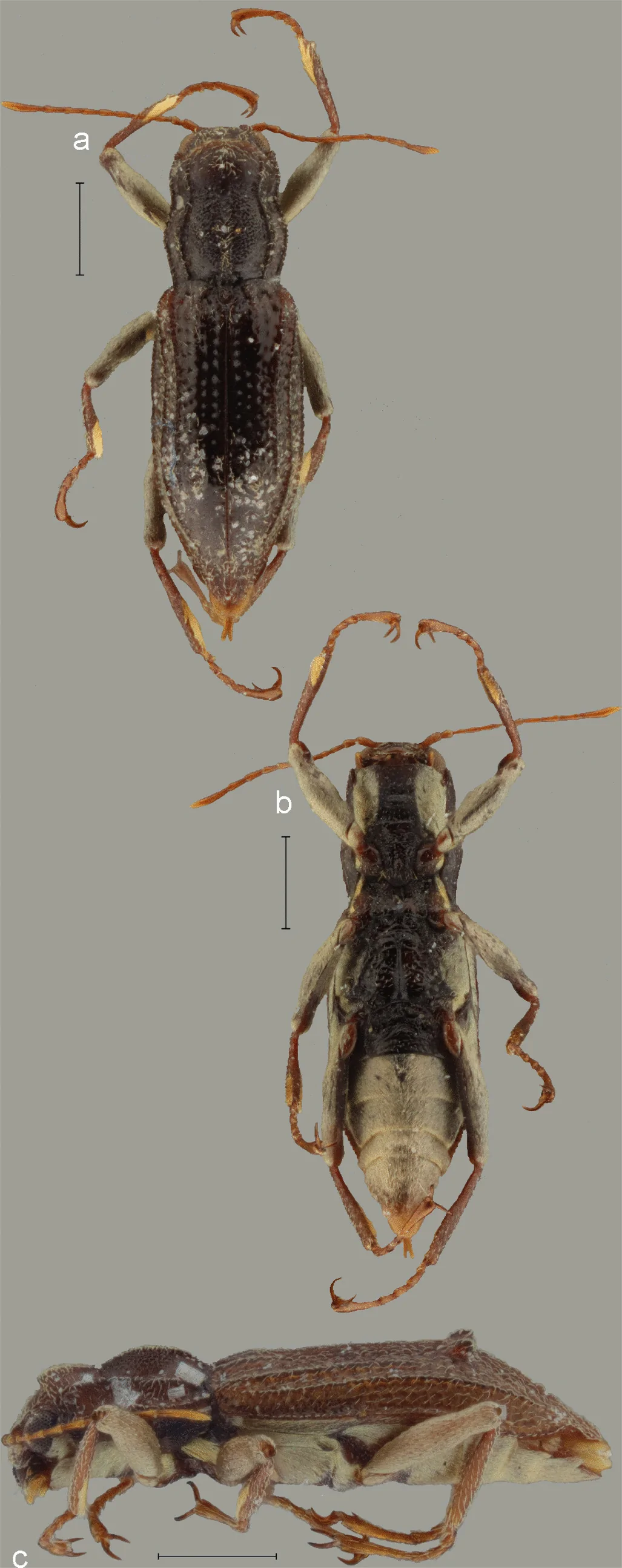
*Microcylloepus dimorphus* **sp. nov.** *Habitus*. Female. **a** Dorsal view. **b** Ventral view. **c** Lateral view. Scale bar = 0.5 mm

**Fig. 5:**
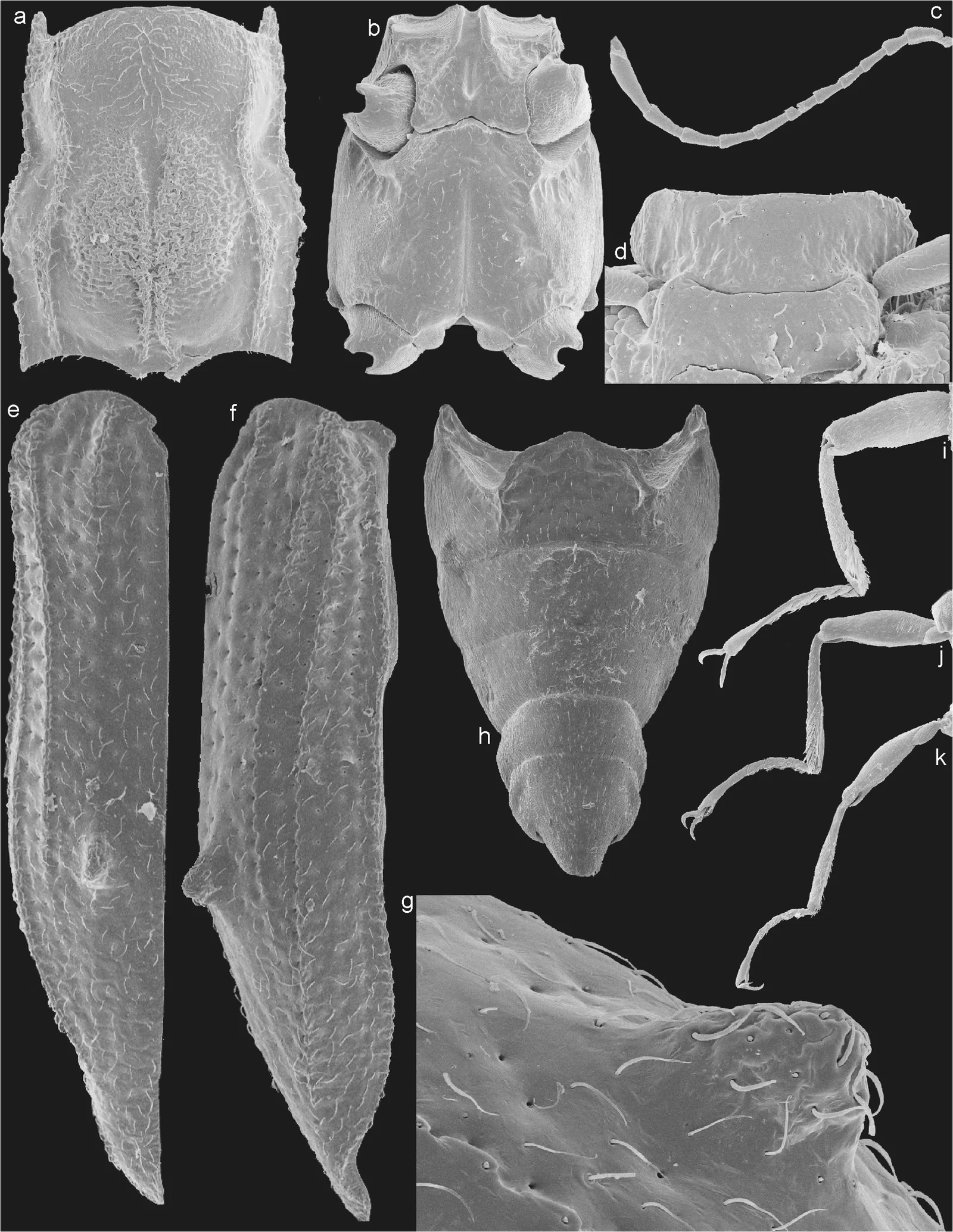
*Microcylloepus dimorphus* **sp. nov.** Female under SEM. **a** Pronotum. **b** Meso and metaventrite. **c** Antenna. **d** Clypeus. **e** Right elytron, dorsal view. **f** Left elytron, lateral view. **g** Tubercle on elytron. **h** Abdomen. **i** Prothoracic leg. **j** Mesothoracic leg. **k** Metathoracic leg

**Fig. 6:**
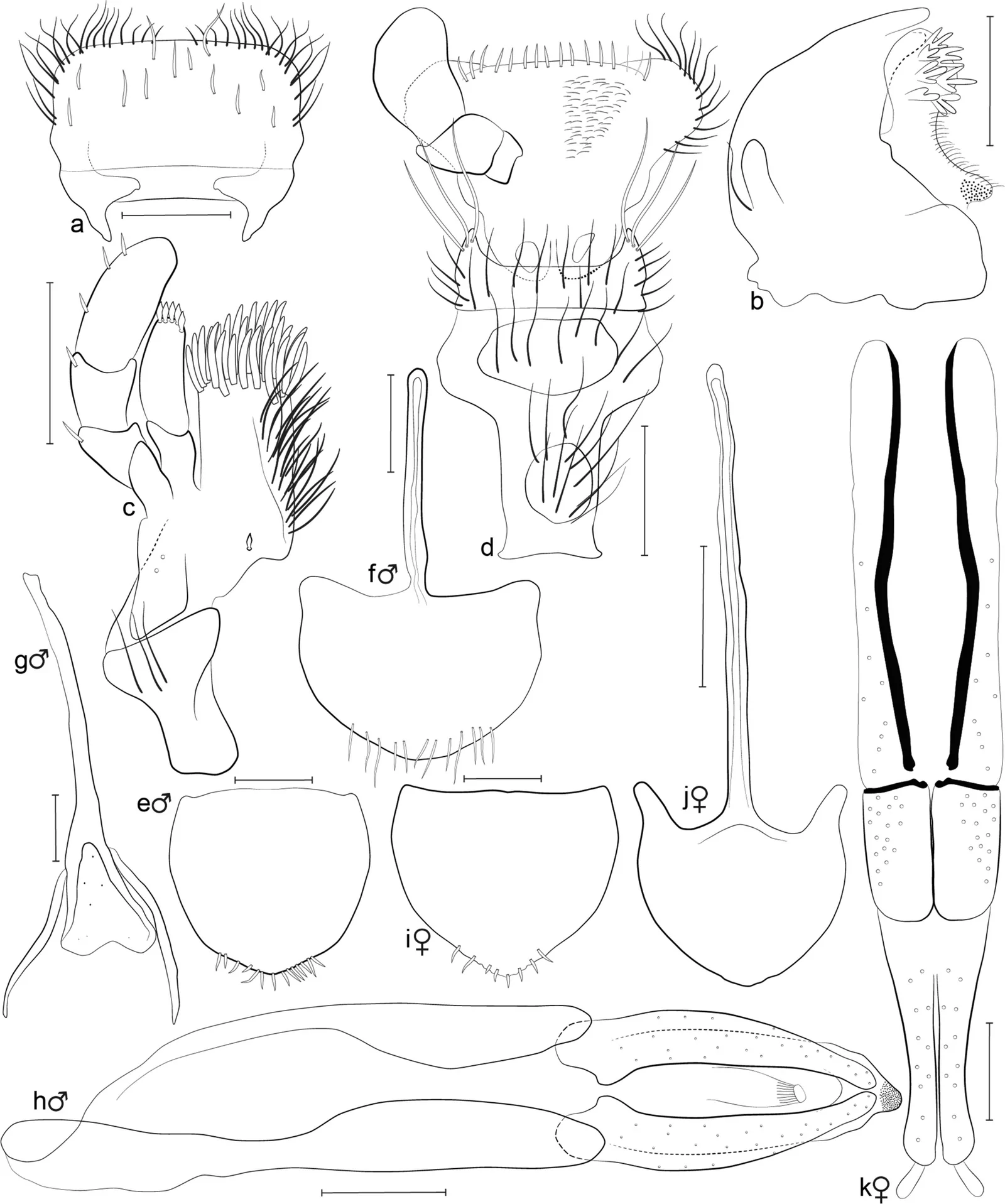
*Microcylloepus dimorphus* **sp. nov. a-h** Male. **a** Labrum, dorsal view. **b** Left mandible, dorsal view. **c** Left maxilla, ventral view. **d** Labium and hypopharynx (right side). **e** Tergite VIII. **f** Sternite VIII. **g** Segment IX, dorsal view. **h** Aedeagus, ventral view. **i-k** Female. **i** Tergite VIII. **j** Sternite VIII. **k** Ovipositor, dorsal view. Scale bar = 0.1 mm

*Adult*: (Figs. 2, 3, 4, 5, 6) Body subparallel, reddish, covered with golden, short, recumbent setae. Elytra with three carinae: on 3rd interval at basal region, on 6th interval from the base to apical 3/5, and on 8th interval extending 4/5 of the elytral length. Male with mesoventrite with a deep mesal cavity to receive prosternal process, posterior region bilobated, strongly prominent like an obtuse callosity; profemur with two deep excavations at middle, forming a prominent tooth; mesofemur with a deep longitudinal sulcus-impression on ventral side, surrounded by fringes of golden setae; mesotibia with a deep longitudinal excavation on distal half, with two tufts of long, dense, golden setae; metafemur with a shallow ventral impression to receive the tibia; abdominal ventrite 2 with a pair of callosities; aedeagus phallobase tubular, longer than penis, parameres arcuated, surround by spatulate penis. Female mesoventrite (Fig. 5b) with posterior region plane, not bilobated, without an obtuse callosity. Legs without excavations, sulci, or any kind of impressions; abdominal ventrite 2 completely covered by tomentum, without callosities; elytron with a tubercle at the apical ¾; coxite elongate, slightly narrowed at apex, apical coxite twice longer than basal.

**Fig. 7:**
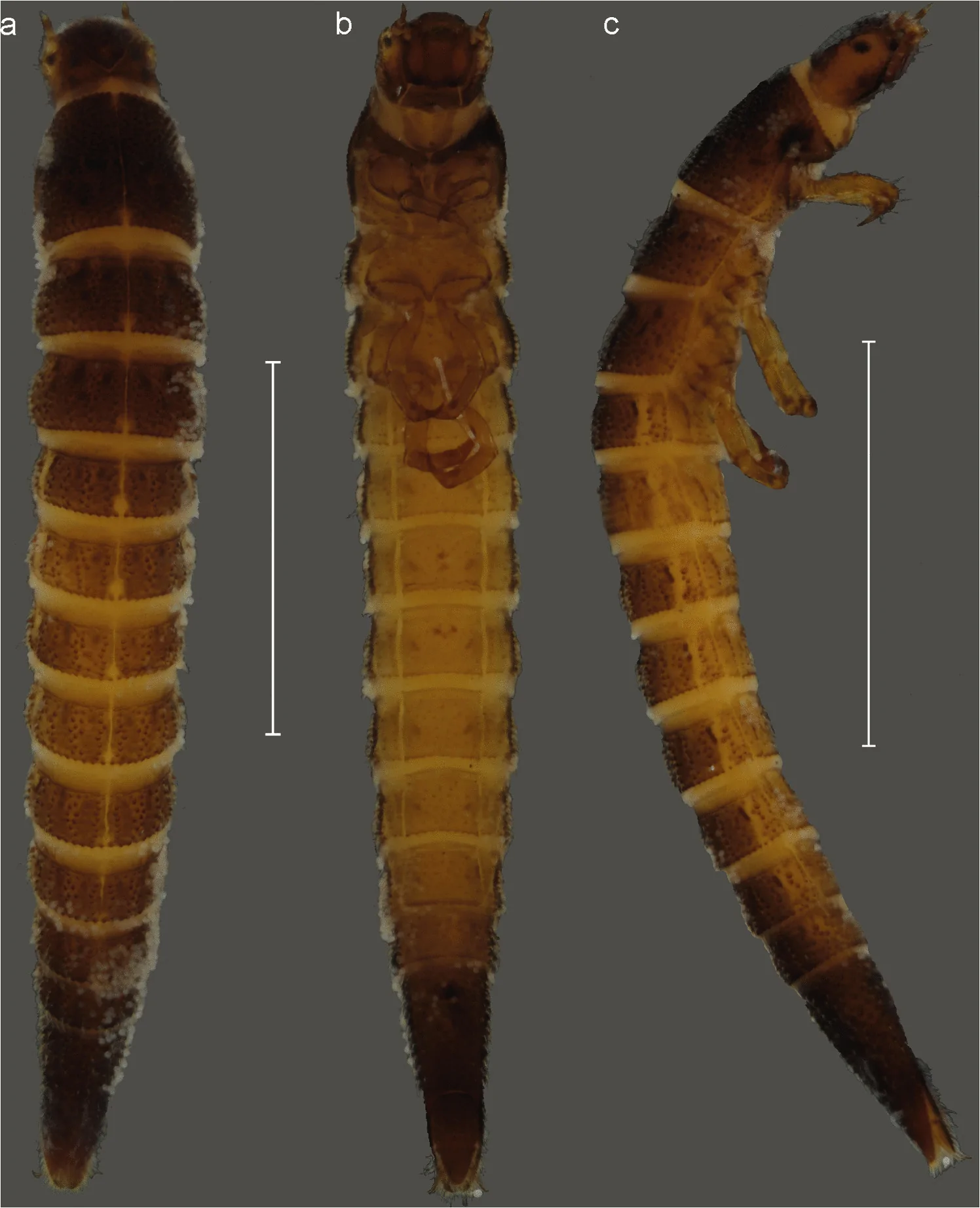
*Microcylloepus dimorphus* **sp. nov.** *Habitus*. Larva. **a** Dorsal view. **b** Ventral view. **c** Lateral view. Scale bar = 0.5 mm

**Fig. 8:**
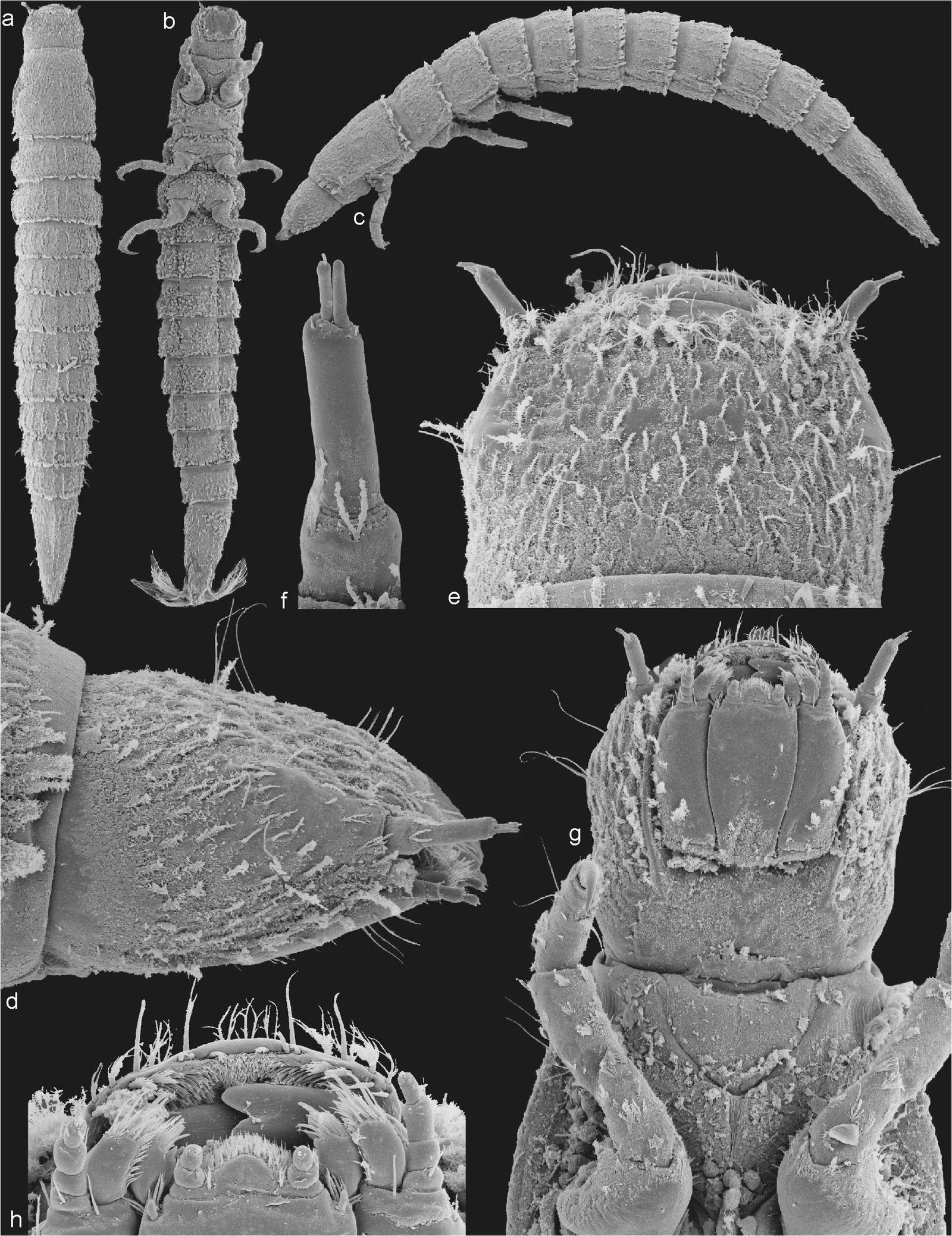
*Microcylloepus dimorphus* **sp. nov**. Larva under SEM. **a** Dorsal view. **b** Ventral view. **c** Lateral view. **d** Head, lateral view. **e** Head, dorsal view. **f** Antenna. **g** Head and prothorax, ventral view. **h** Mouthparts

**Fig. 9:**
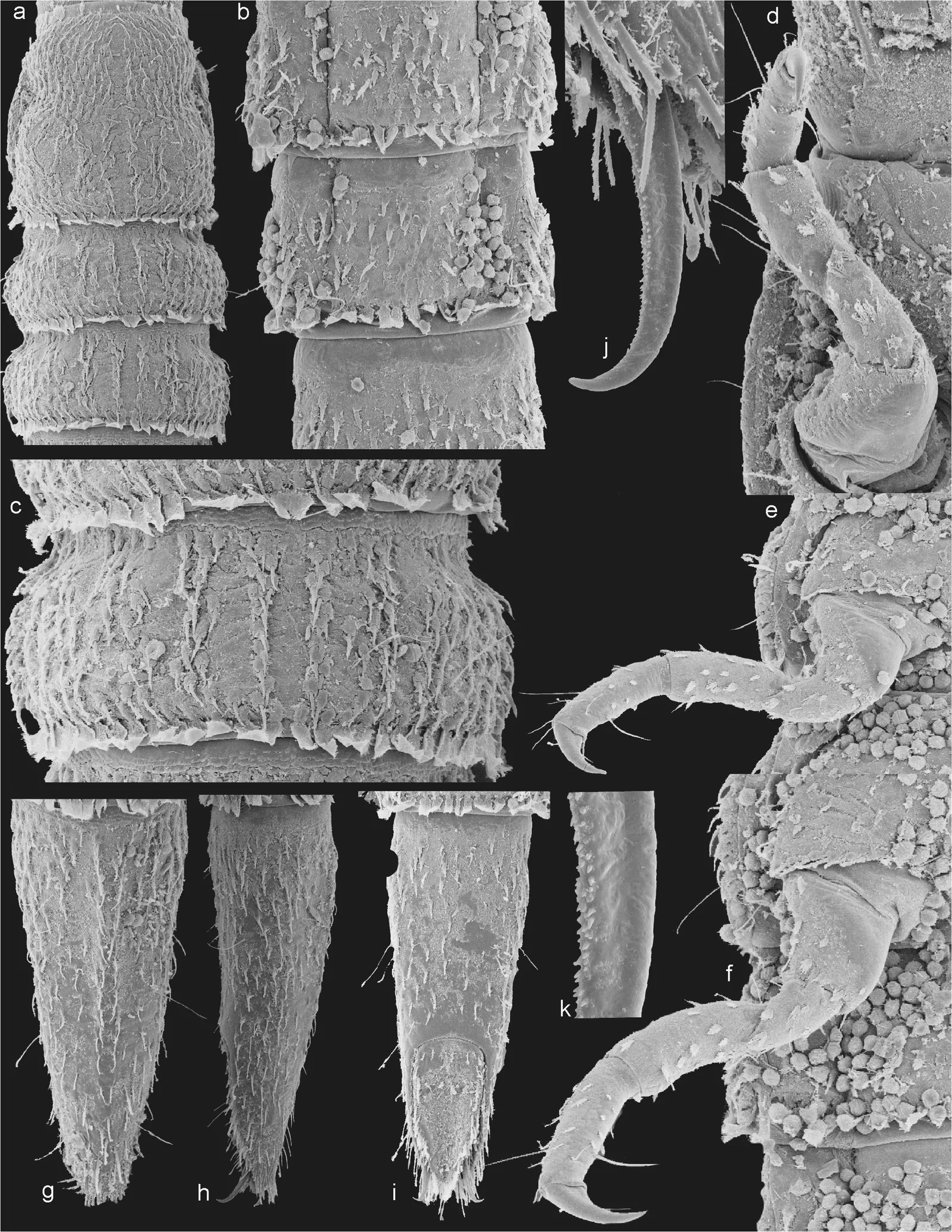
*Microcylloepus dimorphus* **sp. nov**. Larvae under SEM. **a** Thorax, dorsal view. **b** Segment VII and VIII. **c** Segment I. **d** Prothoracic leg. **e** Mesothoracic leg. **f** Metathoracic leg. **g** Segment IX, dorsal view. **h** Segment IX, lateral view. **i** Segment IX, ventral view. **j** Opercular claw. **k** Teeth on opercular claw

**Fig. 10:**
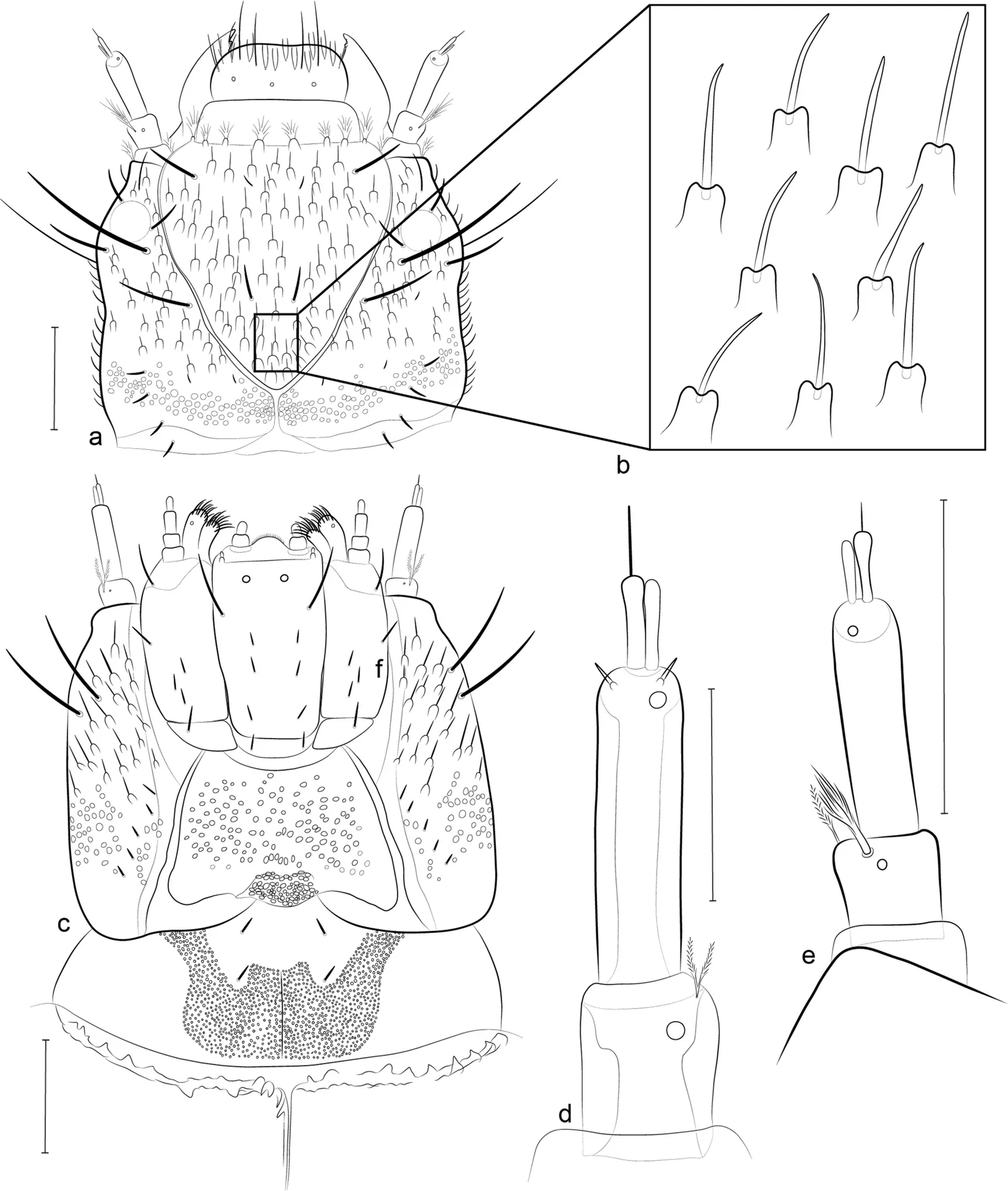
*Microcylloepus dimorphus* **sp. nov**. Larva, head. **a** Head, dorsal view. **b** Head, integument. **c** Head, ventral view. **d** Antenna, ventral view. **e** Antenna, dorsal view. Scale bar = 0.1 mm

**Fig. 11:**
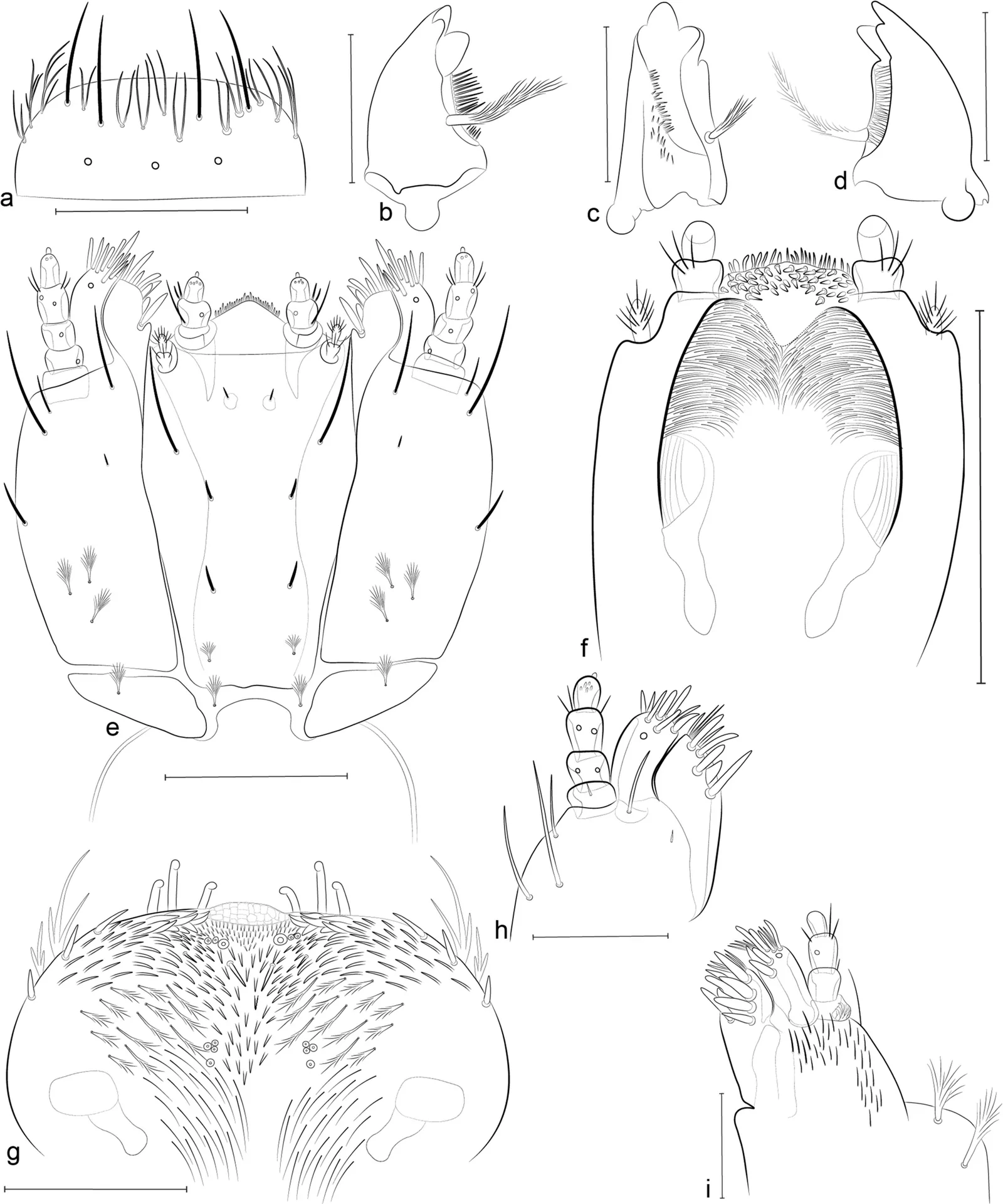
*Microcylloepus dimorphus* **sp. nov**. Larva, mouthparts. **a** Labrum. **b** Mandible, dorsal view. **c** Mandible, internal view. **d** Mandible, ventral view. **e** Maxillolabial complex. **f** Hypopharynx. **g** Epipharynx. **h** Galea and lacinia, ventral view. **i** Galea and lacinia, dorsal view. Scale bar: **a-g** = 0.1 mm. **h-i** = 0.05 mm

**Fig. 12:**
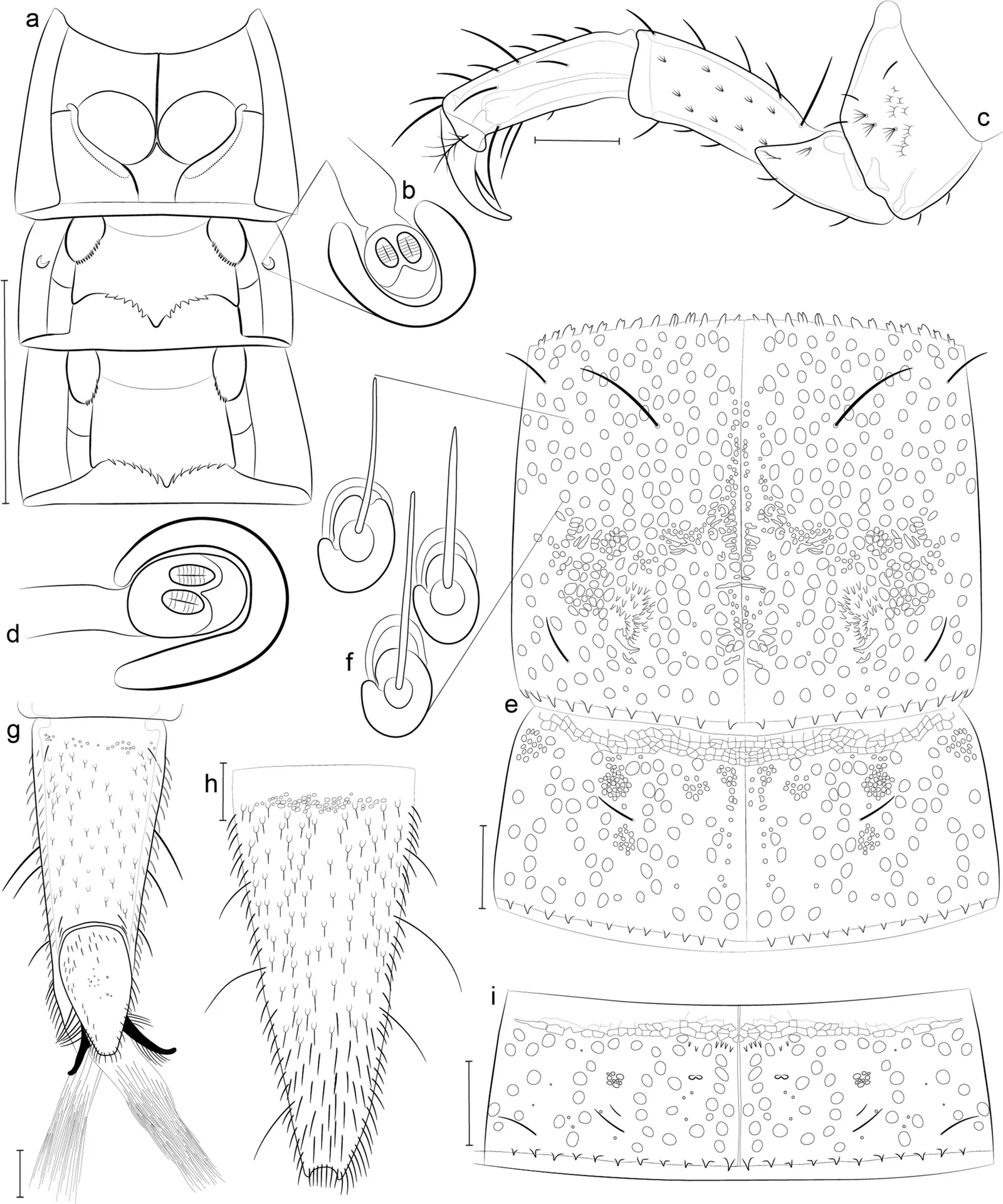
*Microcylloepus dimorphus* sp**. nov**. Larva. **a** Thorax, ventral view. **b** Metathoracic spiracle. **c** Mesothoracic leg. **d** Abdominal spiracle. **e** Pro- and mesothorax. **f** Integument. **g** Abdominal segment IX, ventral view. **h** Abdominal segment IX, dorsal view. **i** Abdominal segment I, dorsal view. Scale bar: **a, c**, **g-i** = 0.1 mm. e = 0.5 mm

*Larva*: (Figs. 7, 8, 9, 10, 11, 12) Coloration reddish-brown, darker at extremities, and dorsally with brown granules; third antennomere almost as long as sensorium; dorsal integument ornamentation similar on thorax and abdomen; anterior angle of mentum with palp-like biarticulate structure; abdominal segment IX 2.6 × longer than operculum; segment IX ventrally with flat setose granules smaller and sparser than dorsal; segment IX with emarginate apex.

***Adult description: Male,*** size range (n = 10): length: 2.30–2.35 mm; width: 0.75–0.80 mm (maximum width of the elytra). Body subparallel, reddish color and covered by golden short recumbent setae dorsally, ventrally darker, covered by plastron laterally.   

*Head.* Partly retractable into prothorax, dorsal surface with micropunctures distanced from each other by the equivalent of their diameter and covered by plastron, without impressions. Eyes protruding laterally (Fig. 2). Frontoclypeal suture almost straight without impressions. Clypeus longer than wide; covering totally the labrum. Labrum (Fig. 6a) subrectangular with long setae on anterolateral margins, surface covered by setae on anterior half. Mandibles symmetrical (Fig. 6b) subtriangular; apex gradually curved mesad, with 2-apical teeth, one thin seta on external lateral lobe; prostheca membranous, with lobes at apex and setae at base. Maxilla (Fig. 6c): cardo elongate, with long setae; stipes subtriangular; palpifer subtriangular; maxillary palpus 4-segmented, apical longest; galea palpiform, 2-segmented, apical segment 1.5 × longer than basal, with a row of stout setae at apex; lacinia subtriangular, with mesal stout setae on inner side and fringe of long, thin setae at apex. Labium (Fig. 6d): mentum flat, subrectangular, covered by long setae; mentum arcuate, converging anteriorly, anterior angles sub-acute and protruding, with three long setae laterally; labial palpus 3-segmented; apical palpomere longer than basal combined; ligula subtriangular, surface in ventral view with setae on disc, anterior margin with stout setae and laterally with long thin setae. Hypopharynx covered by spine-like setae towards disc. Gula subrectangular, covered by long thin setae. Gena covered by tomentum.

*Thorax.* Pronotum (Figs. 2a, 3a) longer than wide; anterior and posterior margins strongly arcuate and lateral margins strongly sinuous and crenulate; strongly constricted on anterior 1/3; complete sublateral carinae raised and strongly sinuous; narrow longitudinal impression on disc deep, oblique impressions on basal 1/3; two prescutellar fovea. Prosternum with anterior margin sinuous, lateral sides densely covered by plastron; prosternal process without plastron, covered by golden setae, with a rounded apex; proepisternum and proepimeron covered by plastron. Hypomeron covered by micropunctures. Elytron (Figs. 2a, 3d, e) elongated, almost 4 × longer than wide, covered by rows of golden setae on intervals; apex rounded; lateral margins crenulate and emarginate; 3rd interval subcarinate on basal region, carinae on 6th interval extending from the base to apical 3/5, carina on 8th interval reaching 4/5 of the elytral length; epipleura narrow, wider anteriorly, covered by plastron (Fig. 3e). Scutellum (Fig. 3b) shield shaped, without punctures, with a few setae. Mesoventrite (Fig. 3c) with a deep mesal cavity to receive prosternal process, posterior region bilobated, strongly prominent like an obtuse callosity; mesepimeron covered by tomentum. Metaventrite twice wider than long, disc glabrous, lateral margins densely covered by plastron; discrimen complete, reaching anterior and posterior margins. Legs covered by tomentum except tarsi. Pro- and mesocoxa rounded, metacoxa transverse. Trochanter subtriangular with thin setae at apex. Profemur (Fig. 2, 3h, l) with two deep excavations at middle, forming a prominent tooth. Protibia (Fig, 3 l) long and thin, 10 × longer than wide, apex gradually curved mesad, with a fringe of golden long setae. Mesofemur (Fig. 3i) with a deep longitudinal sulcus-impression on ventral side, surrounded by fringes of golden setae. Mesotibia (Fig. 3k), elongate, thin, with a deep longitudinal excavation on distal half and two tufts of long, dense, golden setae. Metafemur (Fig. 3j) elongate, with a shallow ventral impression to receive the tibiae. Metatibia long and thin. Tarsus elongated, with a few thin setae and short spines on ventral surface; apical tarsomere as long as the preceding four combined. Claws simple and long.

*Abdomen*. (Figs. 2b, c, 3f, g). Covered by plastron and micropunctures. Disc of ventrite 1 glabrous, surrounded by a complete pair of carinae. Ventrite 2 with a pair of callosities (Fig. 3g) on anterior half, disc glabrous. Tergite VIII (Fig. 6e), semicircular, anterior margin slightly sinuous, posterior margin rounded, with setae. Sternite VIII (Fig. 6f), trapezoid, about as long as wide, very narrow and elongate anterior projection, posterolateral margins angulate, posterior margin arcuate, with setae. *Genitalia*. (Fig. 6h), Phallobase tubular, 3 × longer than wide, longer than penis, asymmetric. Parameres arcuated, surrounding the penis on basal 4/5, with sensorial pores. Penis spatulated, twice wider at the middle, apex rounded with sensorial pores, fibula present and corona crow like shape.

*Female:* Strongly sexually dimorphic. Slightly longer than males, size range (n = 10): 2.5–2.55 mm; width: 0.75–0.80 mm. Disc of pronotum more punctate than in males (Fig. 5a). Mesoventrite (Fig. 5b) with posterior region plane, not bilobated, without obtuse callosity. Legs without excavations, sulcus, or any kind of impression (Fig. 4). Ventrite 2 completely covered by tomentum, without callosities. Elytra with a tubercle (Figs. 5f-g) on apical ¾. Tergite VIII (Fig. 6i) nearly pentagonal-shaped, covered with short setae at apex, nearly straight margin, slightly angulate lateral margins, broadly rounded posterior margin. Sternite VIII (Fig. 6j) arrow-head shaped, about twice longer than wide, anterior projection very long, posterolateral margins strongly prominent, posterior margin rounded. Ovipositor symmetrical (Fig. 6k). Valvifer membranous, ca 3x longer than coxites, subparallel; baculus sclerotized, bar-like. Coxites elongate, slightly narrowed at apex; apical coxite twice longer than basal; apices parallel; surface with sensorial pores. Styli slightly curved and divergent.

***Comparative notes: Microcylloepus dimorphus*** **sp. nov.** is completely different from all other *Microcylloepus* species and is easily recognized by high sexual dimorphism. The shape of the male’s legs, ventrite 2 with a pair of callosities, and the female elytra with a tubercle on the apical ¾ is unique in the genus.

***Larva description:*** Length: 1.8–2.0 mm; width: 0.7–0.8 mm. Body elongate, fusiform, convex dorsally and flattened ventrally (Figs. 7, 8a-c). Reddish-brown with thorax and abdominal apex darker; dorsally with some lighter patches and a pale longitudinal line; intersegmental areas cream-colored to translucent. Ventral integument brownish-yellow with legs and thorax lighter reddish-brown; segments VIII–IX darker. Integument with granules dorsally and on pleurites of thoracic and abdominal segments, each with one seta; pubescence brush-like with some simple and longer setae; surface with glabrous areas. Ventral integument with setose granules sparser.

*Head* prognathous (Figs. 8d, g), slightly longer than wide, visible dorsally; lateral margins slightly sinuous. Epicranial suture Y-shaped (Fig. 10a), slightly convergent distally; coronal suture short. Head dorsally covered by elongate darker granules, each with an apical seta (Figs. 8e,10a, b), sparser laterally; granules with brush-like setae anteriorly and simple on remaining areas; frontal area with three pairs of simple setae (Fig. 10a): one anteriorly, near frontal angles, one at middle near frontal base, and one outside of each frontal arm; each side with six setae around each stemma; frontoclypeal suture straight; frons with transverse row with four pairs of brush-like setae near frontoclypeal suture; one brush-like seta on each side of frons, near antennifer; smaller flat rounded tubercles near base of head; basal area with four short and wide setae arranged in an inclined row on each side; laterally with short curved spiniform setae. One dark stemma on each side formed by cluster of stemmata (Fig. 7). Ventrally (Figs. 8g, 10c), head with setose granules sparser than dorsally; brush-like setae near maxillolabial complex (Fig. 10c); two, long, simple setae on each side, below each stemma; many small darker flat rounded tubercles near base, especially at gular area; each side with four short and wide setae arranged in an inclined row outside each gular line. Antenna (Figs. 8f, 10d, e) as long as mandibles, cylindrical with three antennomeres; antennifer membranous and band-like; first antennomere slightly longer than wide; distal margin with one lateroexternal simple seta, one dorsal brush-like seta, two campaniform sensilla; second antennomere three times longer than wide, bearing at apex, third antennomere and one narrow and elongate membranous sensorium, side by side; apical region of second antennomere dorsally with a rounded sensorium and ventrally with two tiny setae (one longer) and one short sensorium (Fig. 8f); third antennomere elongate, narrow, reduced, slightly longer than distal sensorium and with one seta at apex. Clypeus membranous, transverse. Labrum (Fig. 11a) transverse with lateral margins and fore angles continuous and rounded; densely covered by setae on distal half, with four long and simple setae, and about 13 bifid or brush-like setae. Epipharynx (Figs. 8h, 11g) with three wide and short setae on each side; simple and brush-like setae directed towards middle; tiny setae at middle, below anterior margin, with three sensilla on each side; each side, near middle with a group of three campaniform sensilla, and one sensillum below each group. Mandibles (Figs. 11b-d) elongate with three rounded teeth; ventrally, penicillus well developed, with a ventral fringe of simple setae; articulated setose, median, long process projecting mediad. Maxillolabial complex (Figs. 8g, 11e), almost quadrangular. Maxillae (Fig. 8h): cardo transverse, subtriangular with one short seta; articulatory membrane absent; stipes elongate, subrectangular and undivided, with seven setae (one longer on each side of palpus); galea and lacinia separate (Figs. 8h, 11h-i); lacinia longer, curved and narrower than galea; apices of galea and lacinia (Fig. 8h) with wide and long setae, more concentrated dorsally; simple setae apically. Dorsally, stipes with simple, moderately short setae below palpus and lacinia; two brush-like setae laterally. Maxillary palpi (Figs. 11e, h-i), with 4 palpomeres: first palpomere transverse, ventrally with one short and wide seta, and dorsally with lateral brush-like setae; second palpomere almost as long as wide, ventrally with two campaniform sensilla; third palpomere longer than wide, ventrally with two campaniform sensilla and dorsally with one campaniform sensillum, and three setae near apex (two lateral externa and one lateral interna); fourth palpomere elongate, narrower than third, with several apical short membranous sensilla (one longer). Labium (Figs. 8h, 11e) elongate, wider than maxilla; prementum transverse; ligula membranous, prominent and rounded; apex densely covered by tiny and stout setae; mentum elongate, subrectangular with four pairs of setae (one longer) and one pair of campaniform sensilla; each anterior angle of mentum with mental palp-like biarticulate structure articles; two setae below postmentum, between cardines. Labial palpi (Fig. 8h) with two palpomeres; palpiger band-like, partially membranous; first palpomere slightly wider than long, ventrally with one laterobasal seta and one campaniform sensillum; dorsally with three laterodistal setae; second palpomere narrower than first, elongate with sensilla at apex (one longer). Hypopharynx (Fig. 11f): anterior area continuous with ligula, margined by tiny and stout setae; subapical area raised and covered by thin setae inwardly directed. Gular sutures present; gular area wide, partially covered by flat rounded tubercles. Cervical region membranous with two darker areas (Fig. 10c), covered by tiny flat granules, forming W-shaped darker area; four short setae.

*Thorax* and abdomen with semicircular granules, each with one seta [Figs. 12e, (only density of setose granules represented; setae not included)]. Prothorax (Figs. 7a, 9a, 12e) slightly wider than long, margined anteriorly and basally by irregular teeth; ventrally with 5 sclerites (Fig. 12a). Meso- and metathorax (Figs. 7a, 9a) with a band of flat subquadrangular sclerites at anterior margin; posteriorly margined by teeth; ventrally with three sclerites on each side (Fig. 12a). Mesothorax (Figs. 12a, e) ventrally with lateral spiracle; spiracle opening at apex of small tubercle (Fig. 12b). Pronotum elongate, wider than head, slightly narrowed anteriorly; slightly convex; midline present; covered by darker granules of several sizes, each with one apical seta (represented by marks in Fig. 12e), and also groups of microspines and simple setae; each side with three long simple setae (two lateral); some areas with denser smaller and/or irregular flat granules on each side, below middle; posterior area with darker elongate flat granules, each with an apical brush-like seta. Pleural area (Fig. 9d) densely covered with darker, elongate, flat granules, each with an apical seta; two, long, simple setae near base and two, shorter laterally. Accessory sclerite present. Postpleurite with sparse, setose, flat granules and one long, simple seta near procoxal cavity. Posterior sternum prominent anteriorly between procoxae; procoxae separated by one sclerite; anterior margin tuberculate. Meso- and metanotum (Fig. 9a) almost same length; metanotum slightly narrower; both with midline and covered with darker elongate granules, each with an apical seta; each side with one simple dorsal and two lateral longer setae; anteriorly with a transverse band of small subquadrangular brownish flat tubercles; one (sometimes two) groups of smaller and denser flat granules on each side; posterior area with darker elongate flat rounded tubercles, each with an apical brush-like setae. Meso- and metathorax ventrally with elongate prepleurite (Figs. 9e, f, 12a), subtriangular with posterior margin toothed; pleurite almost glabrous; meso- and metaventrite prominent between coxae. Legs (Figs. 7b-c, 9d-f, 12c) long, increasing in length from pro- to metatothoracic; with simple and brush-like setae; coxa inserted at midline of thorax, transverse to slightly elongate, subtriangular, with small tubercles lateroexternally near base; trochanter subpentagonal, ventral margin longer and with three setae; two ramified setae near anterior margin; femur elongate with eight setae at dorsal and three at ventral margin; tibia narrower than femur with many setae, majority simple setae; pretarsus with one basal seta.

*Abdomen* (Figs. 7, 8a-c) long, with 9 segments; segments I–VIII band-like, decreasing in width and slightly increasing in length to apex; dorsally with midline and covered with darker rounded granules, each with one apical seta; distal margin with band of darker elongate teeth (Figs. 7a, 9c), each with an apical brush-like seta; dorsally, each half with three simple setae (laterointernal longer); biforous spiracles on segments I–VIII (Fig. 12d); tracheae with large aerial sacs; pleural sclerites on segments I–VII (Figs. 7b-c, 8b, 9b). Segment IX elongate (Figs. 9g-I, 12g-h), slightly longer than three preceding segments together, gradually narrowed apicad; apex emarginate with fringe of setae (Fig. 12h); ventrally with apical operculum and two opercular claws (Figs. 9i, 12h); claws with several small teeth on internal margin (Figs. 9j-k); operculum 0.3 × sternite length, slightly narrowed apicad with apex widely rounded; retractile tuft of tracheal anal gills present (Fig. 12g). Integument dorsally covered by granules each with one apical simple or brush-like seta (Fig. 12h); longer setae near lateral margins; a band of flat subquadrangular tubercles at base and simple setae near apical third; ventrally, with flat setose granules smaller and sparser.

***Comparative notes:*** Comparing the larva of *Microcylloepus dimorphus* **sp. nov.** with larval description of *M. inaequalis* (from Hinton 1940a) it was verified that both species present: one stemma on each side; antenna with three antennomeres; mandibles with three obtuse teeth; penicillus well developed; articulated setose median long process; maxillary palpi with four palpomeres; galea and lacinia separate with wide setae at apex; labial palpi with two palpomeres; mentum undivided; stipes with differentiation of palpifer; hypopharynx with sclerotized rod on each side; prothoracic, meso- and metapleura divided in two parts; segment VIII forming a complete ring; apex of segment IX arcuate and emarginate; terga of thoracic and abdominal segments with the granules for the most part similar to those of head, but usually as coarse as those of anterior row; anterior area of all segments except pronotum with band of fine flat-topped, subquadrate to round tubercles; tubercles of sternites sparser than those of tergites and not arranged in rows; pretarsus with one basal seta; posterior margin of all segments except IX and anterior margin of pronotum with a row of dense dentiform tubercles; spiracles opening on apices of small tubercles.

However, the larva of *Microcylloepus dimorphus* **sp. nov.** can be distinguished by its reddish-brown body coloration, with the thorax and abdominal apex darker; prementum with palpiger; gula distinct; head surface densely pubescent; clypeus distinct; stipes with palpifer; pronotum and abdominal segments lacking granules partially arranged in longitudinal rows; tergite IX without a median longitudinal ridge; and all spiracles smaller than the mesothoracic spiracle. In contrast, *M. inaequalis* presents a dark-brown to brownish body coloration; prementum without palpiger; gula indistinct; head surface sparsely pubescent; clypeus not distinct; stipes without palpifer; pronotum with granules on the posterior half partially arranged in irregular longitudinal rows; tergites of the last two thoracic and first eight abdominal segments with granules arranged in similar rows; tergite IX with a strongly convex median longitudinal ridge; and all spiracles similar in size to the mesothoracic spiracle.

***Etymology:*** The specific epithet “*dimorphus”* refers to the strongly sexual dimorphism between males and females.

***Distribution:*** Brazil (Mato Grosso do Sul State).

***Sexual dimorphism:*** Sexual dimorphism in Elmidae is not common and has been reported in a few Neotropical taxa. Hinton (1940a, 1940b) discussed sexual dimorphic characteristics into *Cylloepus* Erichson 1847, *Gyrelmis* Hinton 1940a, 1940b, and *Xenelmis* Hinton 1936. Recently was reported in some species such as *Neoelmis guarani* Shepard and Barr 2016; *Cylloepus dimorphus* Shepard et al. 2021; *Amazonopsis* Barr 2018; and *Xenelmis* species (see Fernandes et al. 2023). However, this represents the first record of the condition in *Microcylloepus*. Extreme morphological differences between males and females are hardly ever, and presently only *Neoelmis guarani* has a similar pattern of dimorphism as is found in *M. dimorphus* **sp. nov.**

***Mating behavior:*** Copulation was not observed between individuals in the samples, but we can speculate on a possible scenario based on the morphological structures of males and females. As reported by Shepard and Barr (2016), the female elytral tubercles probably fit on pair of callosities of male abdominal ventrite 2. The male mesoventrite with a strongly prominent obtuse callosity should fit on the female pronotal/elytral junction. Finally, the modified male legs with excavations/sulci could be useful to secure the female legs.

*Hexacylloepus misteriosa* **sp. nov.**

**Fig. 13:**
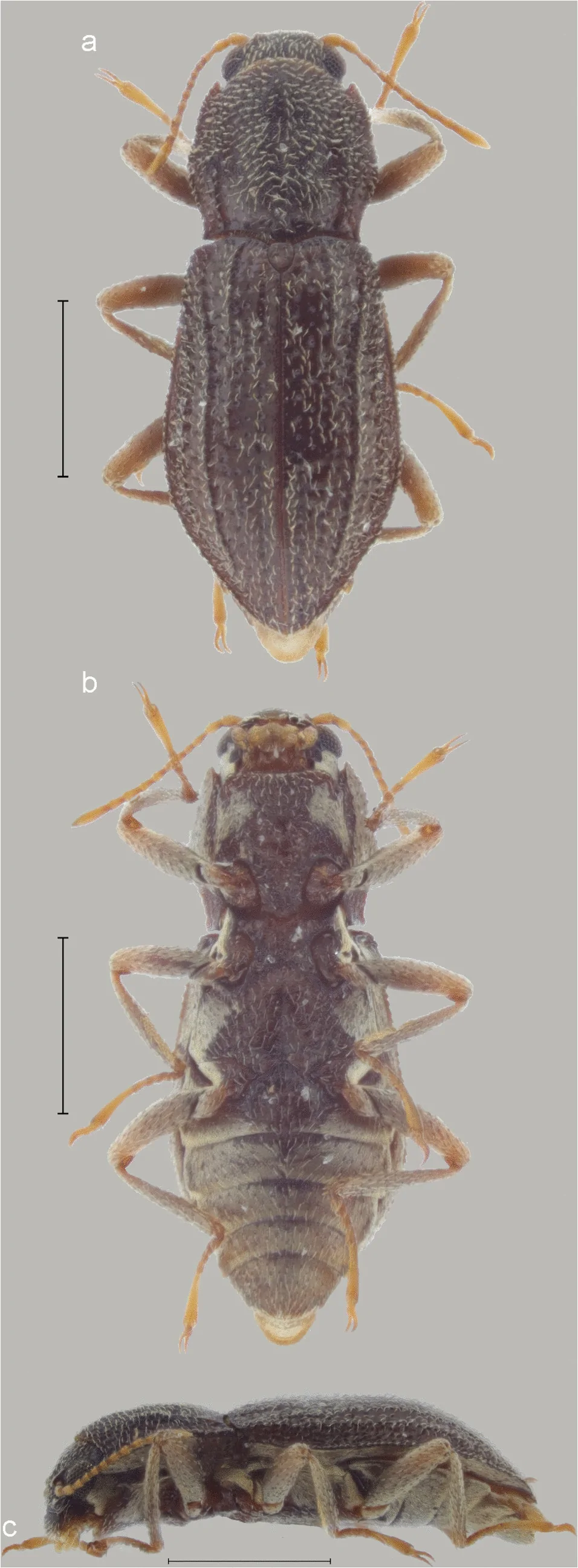
*Hexacylloepus misteriosa* **sp. nov.** *Habitus*. Male. **a** Dorsal view. **b** Ventral view. **c** Lateral view. Scale bar = 0.5 mm

**Fig. 14:**
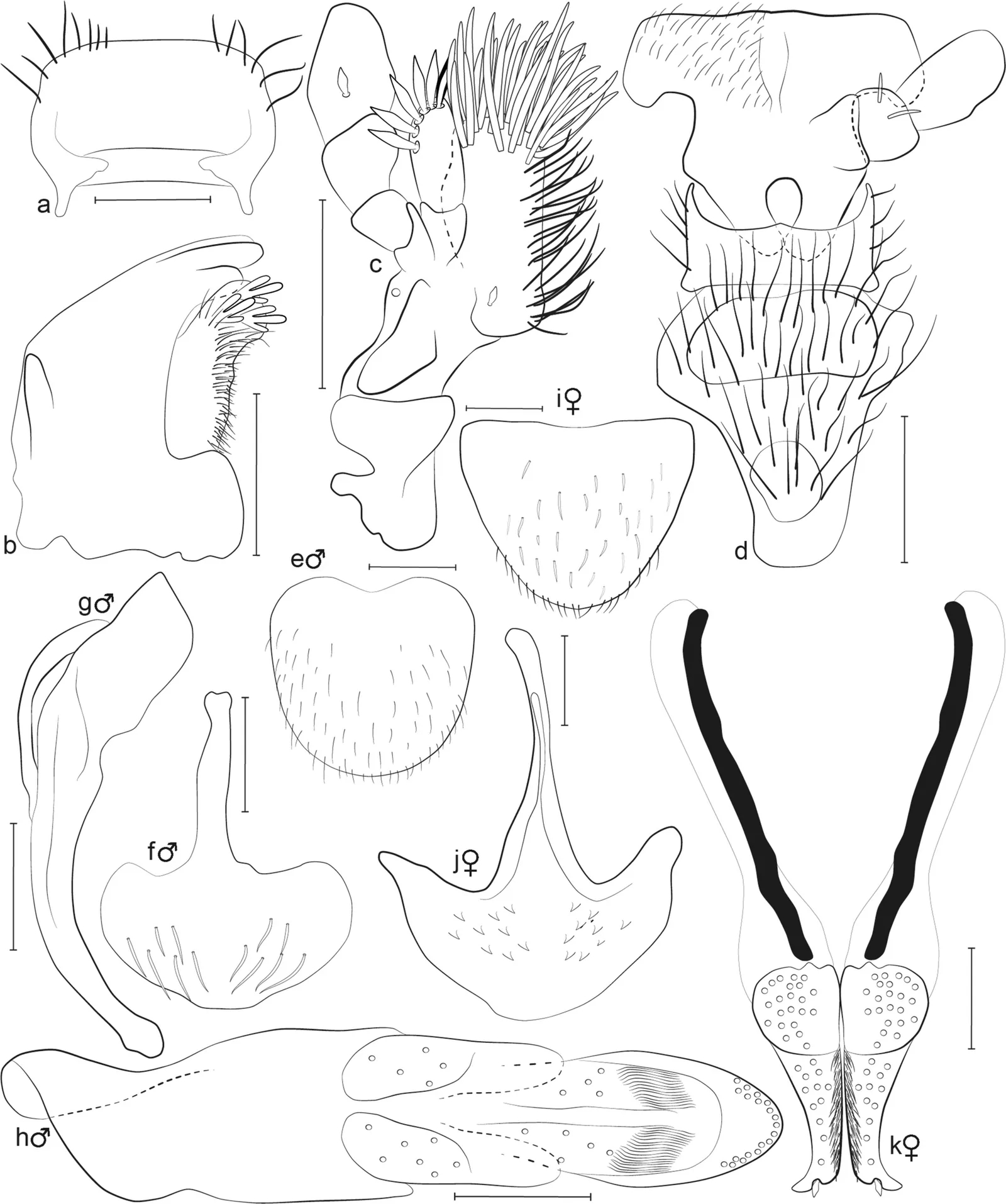
*Hexacylloepus misteriosa* **sp. nov. a-h** Male. **a** Labrum, dorsal view. **b** Left mandible, dorsal view. **c** Left maxilla, ventral view. **d** Labium and hypopharynx (right side). **e** Tergite VIII. **f** Sternite VIII. **g** Segment IX, dorsal view. **h** Aedeagus, ventral view. **i-k** Female. **i** Tergite VIII. **j** Sternite VIII. **k** Ovipositor, dorsal view. Scale bar = 0.1 mm

**Fig. 15:**
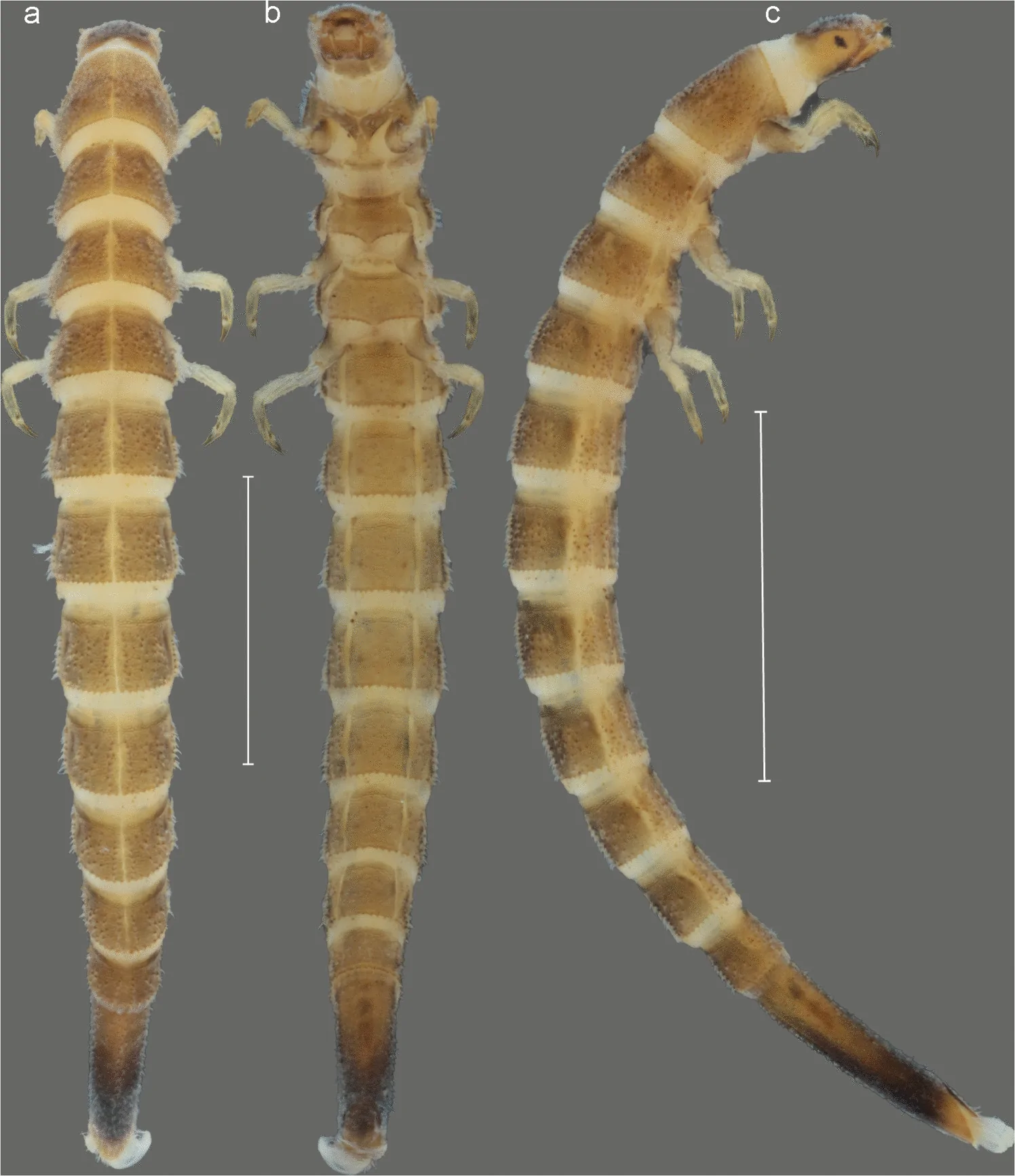
*Hexacylloepus misteriosa* **sp. nov.** *Habitus*. Larva. **a** Dorsal view. **b** Ventral view. **c** Lateral view. Scale bar = 0.5 mm

**Fig. 16:**
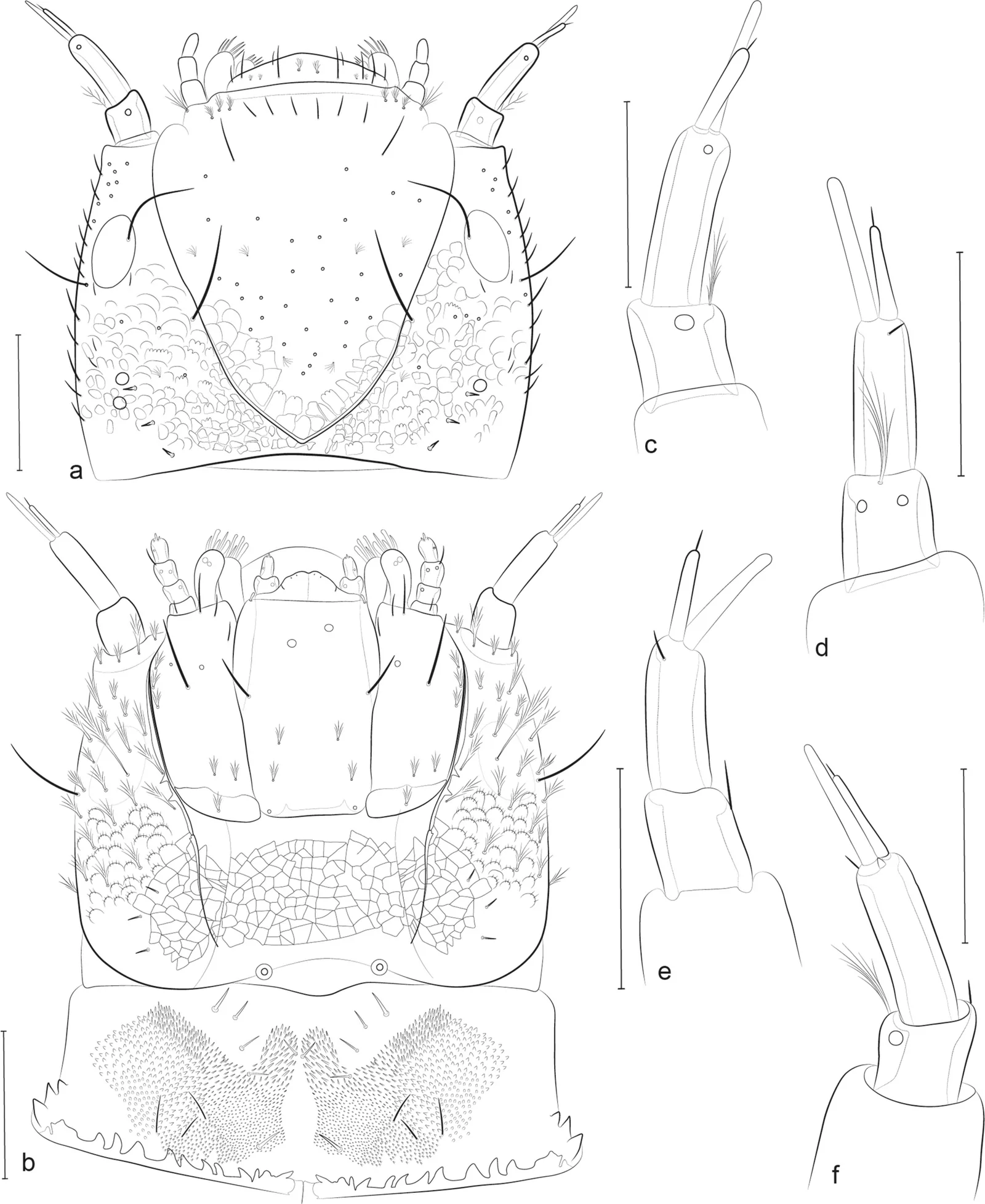
*Hexacylloepus misteriosa* **sp. nov.** Larva, head. **a** Head, dorsal view. **b** Head, ventral view. **c** Antenna, dorsal view. **d** Antenna, lateral view. **e** Antenna, lateral view. **f** Antenna, ventral view. Scale bar: **a-b** = 0.1 mm, **c-f** = 0.05 mm

**Fig. 17:**
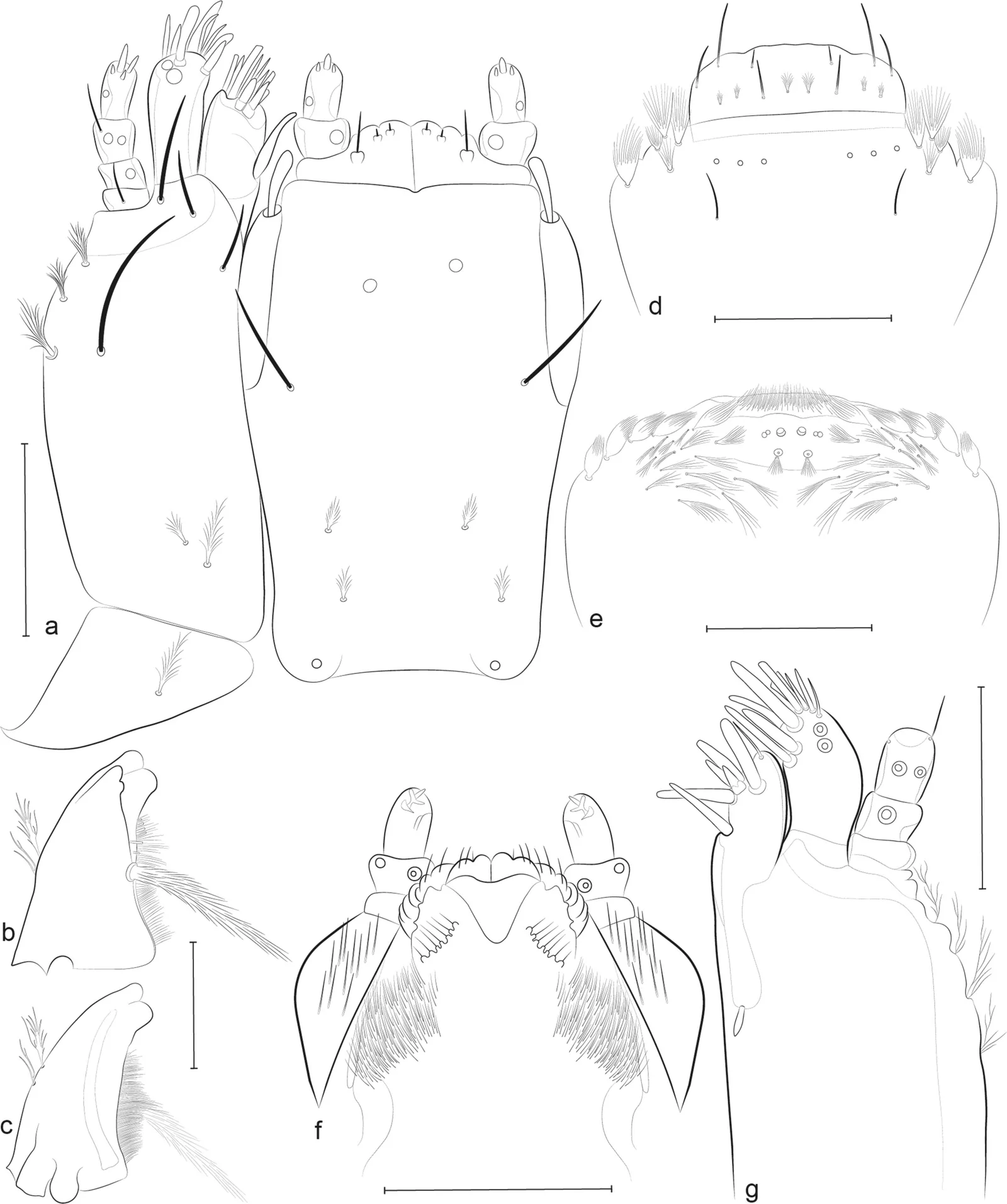
*Hexacylloepus misteriosa* **sp. nov.** Larva, mouthparts. **a** Maxillolabial complex. **b** Mandible, dorsal view. **c** Mandible, ventral view. **d** Labrum. **e** Epipharynx. **f** Hypopharynx. G Galea and lacinia, dorsal view. Scale bar: = 0.05 mm

**Fig. 18:**
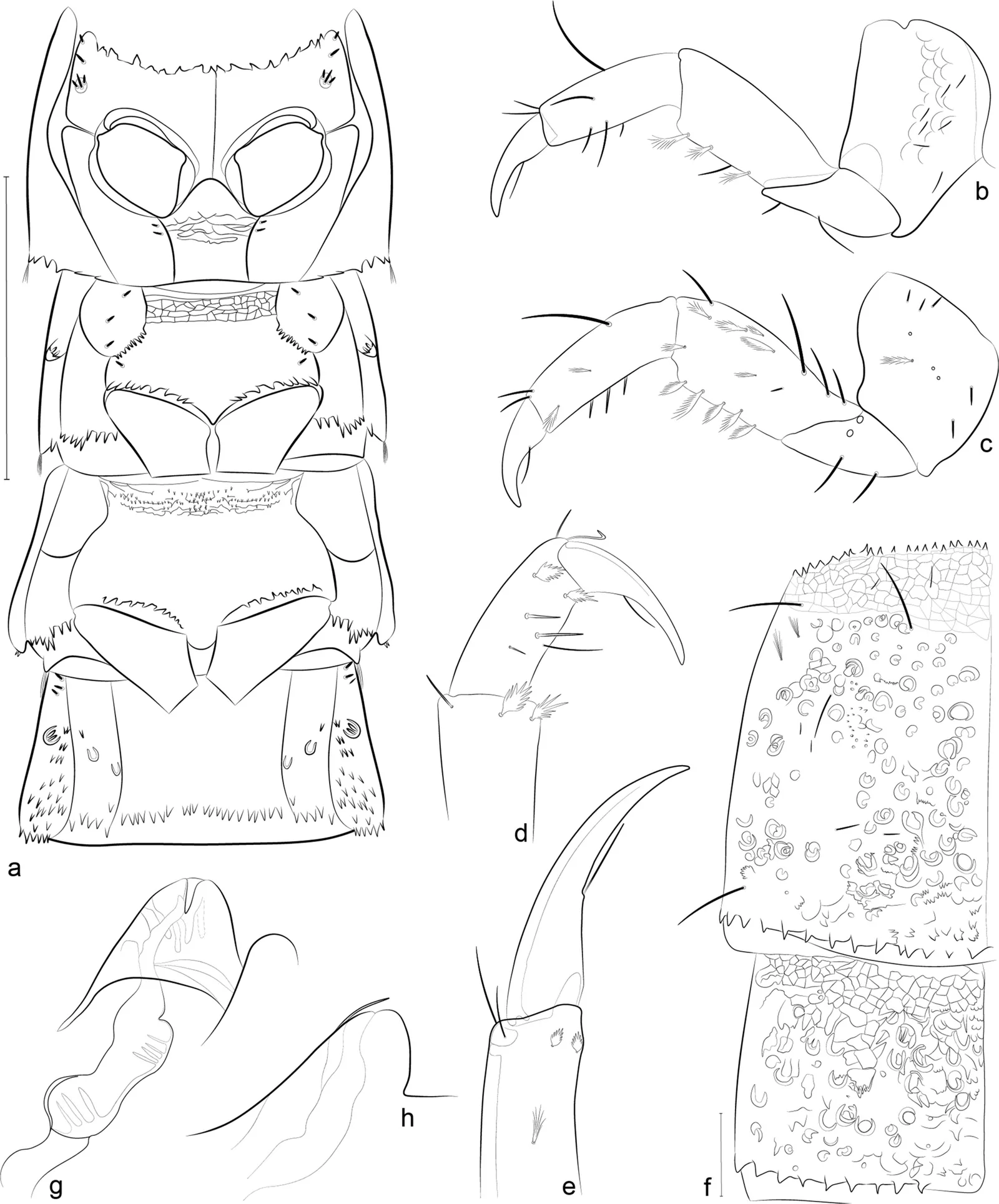
*Hexacylloepus misteriosa* **sp. nov.** Larvae. **a** Thorax and ventrite 1, ventral view. **b** Prothoracic leg, dorsal view. **c** Prothoracic leg, ventral view. **d** Protibiae, ventral view. **e** Protibiae, lateral view. **f** Pro and mesothorax, integument. **g-h** Metathoracic spiracle. Scale bar: **a-c**, f = 0.1 mm. **d-e** = 0.05 mm

**Fig. 19:**
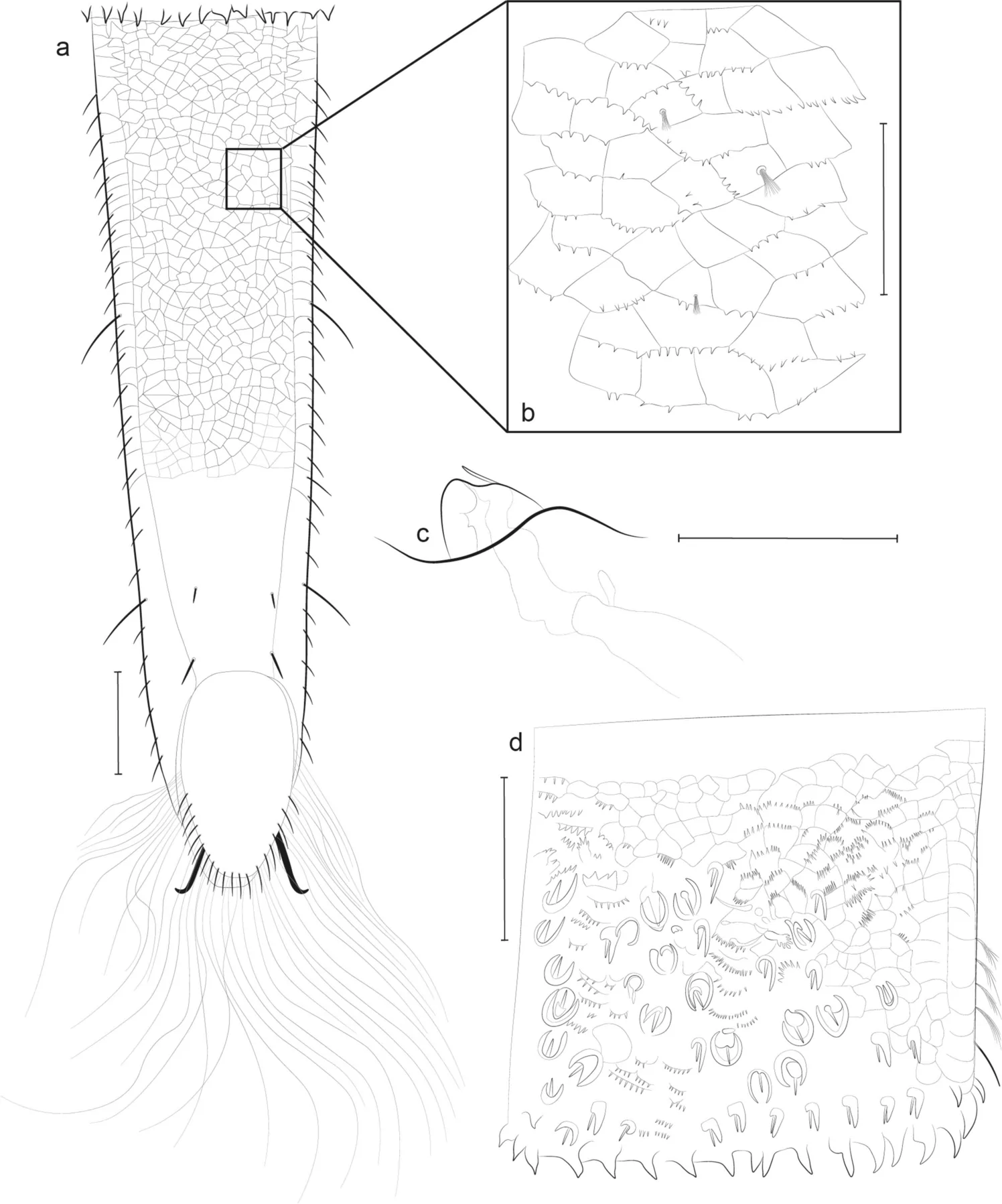
*Hexacylloepus misteriosa* **sp. nov.** Larvae. **a** Abdominal segment IX, ventral view. **b** Integument. **c** Abdominal spiracle. **d** Abdominal segment II, dorsal view. Scale bar: **a, d** = 0.1 mm. **b, c** = 0.5 mm

Figures 13, 14, 15, 16, 17, 18, and 19.

***ZooBank Registration Number:*** https://zoobank.org/NomenclaturalActs/e7ec6b1a-5166-4719-ae6d-e6271570da19

***Material Examined:*** Male HOLOTYPE. ♂ BRASIL. Mato Grosso do Sul./Jardim. Lagoa Misteriosa./22.X.2021. E. Neves et al. leg./021°27′36.0’’S 56°27′21.6’’W. (MZSP 71481). **9 PARATYPES**. 1♀ same data as holotype. (MZSP 71482). BRASIL. Mato Grosso do Sul./Jardim. Lagoa Misteriosa./14.XII.2021. E. Neves et al. leg./021°27′36.0’’S 56°27′21.6’’W. (1♂ and 4♀ MZSP 71483–71487). BRASIL. Mato Grosso do Sul./Jardim. Lagoa Misteriosa./25.IV.2022. E. Neves et al. leg./021°27′36.0’’S 56°27′21.6’’W. (1 in CEMT; 1 in INPA; 1 in MZSP 71489). *Additional material*: **7 larvae**: BRASIL. Mato Grosso do Sul./Jardim. Lagoa Misteriosa./25.IV.2022. E. Neves et al. leg./021°27′36.0’’S 56°27′21.6’’W. (MZSP 10379).

*Diagnosis.*

*Adult*: (Figs. 13–14) Body suboval, dark brown, with antenna and legs reddish, covered with short recumbent setae. Elytra with three carinae: on 3rd interval at base, on 6th interval extending from the base to apical 4/5, and on 8th interval extending 3/5 of the elytral length; hypomeron covered by complete belt of tomentum; tibiae with a complete row of spines; mesotibiae with two fringes of golden setae reaching the distal 3/5; metatibiae with a fringe of golden setae on distal half, tarsus with a few thin setae and short spines on ventral surface; disc of abdominal ventrite 1 strongly rugose, surrounded by a complete pair of carinae. Male with the phallobase tubular, as long as penis, asymmetric; parameres surround the penis on basal 2/5; penis spatulate, strongly constricted at middle. Female with apical coxite 1.5x longer than basal, with a dense fringe of setae on internal margin, apex strongly projected.

*Larva*: (Figs. 15–19) Yellowish-brown with segment IX darker, with brown granules; third antennomere shorter than apical sensorium; each anterior angle of mentum with one wide seta; integument ornamentation different on thorax and abdomen; segment IX 4 × longer than operculum; and ventrally segment IX covered with subquadrangular flat tubercles with short setae near margins.

***Adult description: Male*** length: 1.85 mm; width: 0.7 mm (maximum width of the elytra). Body suboval, dark brown, with antenna and legs reddish, covered by short recumbent setae.

*Head* partly retractable into prothorax, dorsal surface with micropunctures distanced from each other by the equivalent of their diameter and covered by short light golden setae, without impressions (Fig. 13a). Eyes protruding laterally. Frontoclypeal suture inconspicuous. Clypeus longer than wide; covering partially the labrum. Labrum (Fig. 14a) subrectangular with long setae on anterolateral margins. Mandibles symmetrical (Fig. 14b) subrectangular; apex gradually curved mesad, with 3-apical teeth; prostheca membranous, with lobes at apex and setae at base. Maxilla (Fig. 14c): cardo elongate, glabrous; stipes subrectangular; palpifer subtriangular; maxillary palpus 4-segmented, apical subequal to the third, with a stout seta; galea palpiform, 2-segmented, apical segment 1.5 × longer than basal, with a row of stout setae at apex; lacinia subtriangular, with mesal stout setae on inner side, a fringe of long, thin setae at apex, and a stout seta at base. Labium (Fig. 14d): mentum flat, subrectangular, covered by long setae; mentum arcuate, converging anteriorly, anterior angles sub-acute and protruding; labial palpus 3-segmented, apical palpomere as long as two basal combined; ligula subrectangular, integument with setae on disc, anterior almost straight, with short thin setae. Hypopharynx covered by spine-like setae towards disc. Gula subrectangular, covered by long thin setae. Gena covered by tomentum.

*Thorax.* Pronotum (Fig. 13a) as long as wide; anterior and posterior margins strongly arcuate and lateral margins arcuate and crenulate, constricted on anterior 1/3; complete sinuous sublateral carinae; longitudinal impression on disc; two prescutellar fovea. Prosternum with anterior margin almost straight, lateral sides covered by plastron; prosternal process with micropunctures, without plastron, with a rounded apex; proepisternum and proepimeron covered by plastron. Hypomeron covered by complete belt of tomentum, reaching the lateral and anterior margins. Elytra (Figs. 13a, c) elongate, almost 3 × longer than wide covered by two rows of golden setae on intervals; apices rounded; lateral margins crenulate and strongly emarginate; 3rd interval subcarinate on basal region, carina on 6th interval extending from the base to apical 4/5, carina on 8th interval reaching 3/5 of the elytral length; epipleura narrow, wider anteriorly, covered by plastron (Fig. 13c). Scutellum (Fig. 13b) shield shaped, without punctures,with setae. Mesoventrite (Fig. 13c) with a mesal cavity to receive prosternal process; mesepimeron covered by tomentum. Metaventrite twice wider than long, disc moderately deep, with long setae, lateral margins densely covered by plastron and punctures; discrimen complete, reaching anterior and posterior margins. Legs covered by tomentum except tarsi. Pro- and mesocoxa rounded, metacoxa transverse, with a stripe of tomentum on posterior margin. Trochanter subtriangular. Femur 3 × longer than wide. Tibia long and slim, with a complete row of spines, mesotibia with two fringes of golden setae reaching the distal 3/5, metatibia with a fringe of golden setae on distal half. Tarsus elongated, with a few thin setae and short spines on ventral surface; apical tarsomere as long as the preceding four combined. Claws simple and long.

*Abdomen*. (Fig. 13b) Covered by plastron, golden short setae, and micropunctures. Disc of ventrite 1 strongly rugose, surrounded by a complete pair of carinae. Tergite VIII (Fig. 14e), rounded, anterior margin sinuous, posterior margin rounded, with setae. Sternite VIII (Fig. 14f), subtriangular, twice longer than wide, anterior projection narrow and elongate, 1.5 × longer than sternite, posterolateral margins angulate, posterior margin arcuate, apex prominent. *Genitalia*. (Fig. 14h), Phallobase tubular, twice longer than wide, as long as penis, asymmetric. Parameres rounded, surrounding the penis on basal 2/5, with sensorial pores at base. Penis spatulated, strongly constricted at middle, apex rounded with sensorial pores, fibula present and corona shape crown like.

*Female:* External morphology similar to male, except the metaventrite is more punctate and plane on females. Tergite VIII (Fig. 14i) subtriangular, covered with short setae on apex; anterior margin nearly straight; lateral margins slightly angulate; posterior margin rounded. Sternite VIII (Fig. 14j) subtriangular, about twice longer than wide; anterior projection long; posterolateral margins strongly prominent; posterior margin broadly rounded; integument with spines. Ovipositor symmetrical (Fig. 14k). Valvifer membranous, ca. 1.8x longer than coxites, subparallel; baculus sclerotized, bar-like. Coxites elongate, slightly narrowed at apex; apical coxite 1.5x longer than basal, with a dense fringe of setae on internal margin, apex strongly projected, surface with sensorial pores. Styli short, with stout setae.

***Comparative notes: Hexacylloepus misteriosa*** **sp. nov.** resembles *Hexacylloepus zaninii* Polizei et al. 2020 based on general shape; diminutive size (less than 2 mm); pronotum with a longitudinal impression restrict to disc; elytra with apex rounded and lateral margins crenulate and strongly emarginate. However, the two species can easily be differentiated by the following characteristics: in *H. misteriosa* **sp. nov.** tibiae with a complete row of spines, and penis spatulate, strongly constricted on middle; while in *H. zaninii* only the metatibia has a row of spines on distal half, and the penis has the lateral margin sinuous and apex moderately sharp. *Hexacylloepus misteriosa* **sp. nov.** also resembles *Hexacylloepus abdominalis* (Hinton 1937) based on aedeagus shape, but it is easily differentiated by the following characteristics: in *H. misteriosa* **sp. nov.**protibiae without a fringe of golden setae, and abdominal ventrite 2 without a tubercle on the disc, while in *H. abdominalis* the protibiae has a fringe of golden setae and the abdominal ventrite 2 has a tubercle on disc.

***Larva description: ***Length: 1.8–2.0 mm; width: 0.7–0.8 mm. Body elongated, fusiform, convex dorsally and flattened ventrally (Fig. 15). Yellowish-brown with segment IX darker; dorsally with a longitudinal pale dorsal line; intersegmental areas cream to translucent. Integument dorsally and pleurites of thoracic and abdominal segments with brown granules; pubescence brush-like with some simple and longer setae; surface with glabrous areas. Ventrally integument with setose granules lighter and sparser. *Head* (Figs. 16a-b), slightly wider than long, with lateral margins slightly curved; epicranial suture U-shaped (Fig. 16a); frons with subquadrangular flat tubercles near base and with many brush-like short setae on remainder areas (represented by punctures in Fig. 16a); a row with six moderately long setae below labrum and one longer below each extremity of row; gena dorsally with small flat tubercles with distal margin rounded or rounded with microteeth, more concentrate below suture, and intermingled by short brush-like and simple setae (several represented by punctures); one well developed black stemma on each side, formed by cluster of stemmata; two long setae on each side near frontal arms: one above each stemma and other below, nearer each arm; an inclined row with three short setae on each side at basal fourth; several short simple or brush-like setae scattered in this area. Ventrally (Fig. 16b), head with one simple long seta on each side, and many brush-like setae of varied sizes; basal half with subquadrangular flat tubercles, some lateral with anterior margin rounded and with microsetae; each side of head with three short simple setae. Cervical region (Fig. 15c) membranous with two darker areas, covered by microteeth, forming W-shaped darker area; five short setae on each side. Gular suture slightly divergent; gular area with flattened subquadrangular sclerites. Antennae (Figs. 16c-f), long, partially membranous with three antennomeres; antennifer membranous; first antennomere slightly wider than long, with one campaniform sensillum dorsal and one ventral; second antennomere elongate, narrower and twice longer than first, bearing one campaniform sensillum dorsal and one lateroexternal short setae; at apex, second antennomere with one short membranous ventral sensorium, and third antennomere and elongate apical sensorium, side by side; third antennomere elongate, ¼ of width and a half of length of second, with one short seta at apex; distal sensorium elongate, membranous, almost as wide and longer than third antennomere. Labrum (Fig. 17d), transverse, band-like with anterior margin slightly sinuous; four pairs of long simple setae (one shorter) and three pairs of short brush-like setae. Epipharynx (Fig. 17e), partially covered with brush-like setae; each side of anterior margin with five wide brush-like setae; many narrower brush-like setae near middle, midwardly directed; a band of simple short setae anteriorly at middle; two large sensilla near middle with a couple of smaller sensilla on each side, forming a transverse row below short anteriorsetae; two larger sensilla below anterior row. Mandibles (Figs. 17b-c), symmetrical, slightly wider basally, curved; apex with three rounded teeth; ventral tooth small and with a small, rounded projection at base; penicillus ventrally and well developed, formed by fringe of long setae; articulated setose process long and projected mediad; two brush-like lateroexternally setae near middle; dorsally densely micropunctuate. Maxillolabial complex (Fig. 17a): Maxillae: cardo elongate, subtriangular with one short brush-like seta; articulatory membrane absent; stipes elongate, subrectangular and undivided, with lateral margin curve with several brush-like setae; stipes with four long setae anteriorly and five brush-like short setae: two near base and three lateral; galea and lacinia (Fig. 17g), separate and elongate; lacinia longer, curve lateroexternal and narrower than galea; apices of galea and lacinia with wide and long setae, more concentrate dorsally. Maxillary palpi (Fig. 17a), with 4 palpomeres: first palpomere transverse, ventrally with one seta; second transverse, ventrally with one campaniform sensillum; third, longer than wide, ventrally with two campaniform sensilla and one lateral seta, and dorsally, one campaniform sensillum; fourth elongate, narrower than third, ventrally with one campaniform sensillum and dorsally with two campaniform sensilla and one apical seta on each side; about four elongate membranous sensilla at apex. Labium (Fig. 17a), elongate, wider than maxilla; prementum transverse with one pedunculate seta on each side near base of palpi; ligula membranous, prominent and sinuose, with four tiny pedunculate setae near apex; mentum elongate, subrectangular with one long seta on each side, near middle; two campaniform sensilla anteriorly and two near base, four brush-like short setae on basal half; each anterior angle of mentum with one wide seta. Labial palpi (Fig. 17a), with two palpomeres; palpiger band-like, membranous; first palpomere slightly wider than long, with one campaniform sensillum ventrally and two dorsally; second narrower and longer than first, elongate with one campaniform sensillum ventrally; short elongate membranous sensilla at apex. Hypopharynx (Fig. 17f): anterior margin sinuose; several subapical short setae; two inclined rows of short setae inserted in small tubercles, and below, each side with area with fine and dense setae.

*Thorax* (Figs. 15, 18a, f). Integument with simple setae and many flat granules (Fig. 18f), majority in U-shaped; prothorax anteriorly margined by small triangular teeth; pro-, meso- and metathorax margined posteriorly by large irregular triangular teeth. Pronotum (Fig. 15a), slightly wider than long; small transversal band anteriorly with subquadrangular flat tubercles and some short setae; two pairs of long setae below anterior area, one among sclerites and one laterally near base; two pairs of short and two pairs of tiny setae near lateral glabrous area. Ventrally (Fig. 18a), prothorax with five sclerites; procoxal cavities closed posteriorly; meso-and metathorax with three sclerites on each side; mesothorax with one biforous spiracle at apex of a tubercle on each side (Figs. 18g-h). Meso- and metathorax similar in size, wider than long; width almost twice length; basally with band of subquadrangular flat tubercles; dorsal- and laterally with C-shaped flat granules and short setae near middle. Legs (Figs. 15, 18b-e), long and 5-segmented, increasing in size from anterior to posterior; coxae inserted near middle; procoxal cavities closed; procoxae separate by one sclerite; coxae elongated, strongly dilated laterally near apex, forming a wide round tubercle; with an area with small scale-like sclerites near middle; trochanter subtriangular elongate, with some brush-like short setae; femur elongate, with many short and wide brush-like setae in both sides, and simple setae at dorsal and ventral margins (one apical wider); tibia elongate, slightly narrower and almost as long as femur, with one row of short and wide brush-like setae near ventral margin, and a row of simple setae ventral; four very long simple setae near apex; pretarsus long, slender, with a small seta on inner margin (Figs. 18d-e).

*Abdomen* (Fig. 15, 18a, 19): Integument dorsally different from thorax, with flat granules U-or L-shaped near middle and toothed-like near base, each with one seta at middle; density of teeth increasing to apex (Fig. 19d); pleural sclerites present on segments I-VII (Fig. 15b); segments I-VIII with one spiracle on each side (Figs. 18a, 19c), located at apex of a tubercle; spiracles smaller than thoracic and decreasing in size to apex. Segment IX (Fig. 19a), elongate, narrowed near apex; apex rounded; ventral with elongate operculum and two opercular claws; operculum slightly narrowed at distal half, with apex widely rounded; operculum 0.25 × sternite length; tufts of retractable tracheal anal gills present; dorsal carina absent. Ventrally (Fig. 19b), segment IX covered with subquadrangular flat granules with short setae near margins.

***Comparative notes:*** Comparing the larva of *Hexacylloepus misteriosa* **sp. nov.** with description of the larva of *H. ferrugineus* (from Brown 1973), it was verified that both species present: body covered with granules; sides of head pale, around and behind stemmata; legs and antennae brownish; one stemma on each side; antennae each with 3 antennomeres; second antennomere longest; third antennomere shorter than first, slender and bearing minute apical seta; third antennomere shorter than apical sensillum; anterior margin of head, between antennae and clypeus, with an inconspicuous tooth on each side; penicillus and articulated setose process well developed; lateral margin of mandibles bearing one or two brush-like setae; stipes maxillary not well differentiate into a palpifer; labium subquadrate; operculum bearing a pair of long, recurved hooks attached dorsal surface and bordered posteriorly with rather long setae; maxillary palp with 4 palpomeres; galea and lacinia separate, each bearing apical tuft of wide setae; labial palp with 3 palpomeres; each anterior angle of mentum with a large mental setae; posterior margin of tergites and sternites and anterior margin of prothorax bordered by dentate tubercles; procoxal cavities closed, separate by one sclerite; meso- and metapleural each divided into two parts; pretarsus long, slender, with a small seta on inner margin; abdominal segments I-VII with pleura bounded by tergo- and sternopleural sutures; segment IX with rounded apex; tuberculate spiracles on mesothorax and segments I-VIII abdominal; pale mid-dorsal line extending from pronotum to segment VIII and pale area widest near middle of each segment, except pronotum; operculum with 3 tufts of long filamentous, retractile anal gills.

However, the larva of *H. ferrugineus* is characterized by a light grayish-brown body coloration; segment IX blackish-brown except for the brownish apex; and lighter patches on the posterior portion of the pronotum and on the sides of the mesonotum, metanotum, and first six abdominal tergites. The stemmata consist of a cluster of about five fused stemma. Segment IX is elongate, at least three times longer than wide, and bears a feeble longitudinal mid-dorsal carina. The operculum is less than one-third the length of segment IX. The prothoracic pleura are undivided, the mandibles are asymmetrical with 3–4 apical teeth, and the frontoclypeal suture is visible in cleared specimens under high magnification. The labrum is broadly arcuate and bears an irregular basal row of brush-like membranous setae. In contrast, *Hexacylloepus misteriosa* **sp. nov.** has a yellowish-brown body coloration; segment IX dark-brown, becoming brownish at both the apex and base; and lacks light patches on the thoracic and abdominal segments. The stemmata form a cluster with an undetermined number of fused stemma. Segment IX is more elongate, about six times longer than wide, and lacks a longitudinal mid-dorsal carina. The operculum is about one-fourth the length of segment IX. The prothoracic pleura are divided, the mandibles are symmetrical with three apical teeth, and the frontoclypeal suture is not visible. Additionally, the labrum bears three pairs of simple setae.

***Etymology:*** The specific epithet “*misteriosa*” is a noun in apposition and refers to the name of the lake (karst sinkhole) where the new species was found. In the Portuguese language, “misteriosa” means mysterious.

***Distribution:*** Brazil (Mato Grosso do Sul State).

## Discussion

With an increase in taxonomic studies on Neotropical riffle beetles in recent years, especially in Brazil, the number of new taxa has been rising steadily. However, knowledge gaps about the fauna of aquatic insects, particularly in the Elmidae, exist in some geographical regions, e.g. the middle west. Until recently, only *Hexacylloepus zaninnii* had been recorded from Mato Grosso do Sul State. This can be explained by the lack of researchers, studies and sampling effort in this region.

After an intensive one-year survey of Lagoa Misteriosa, a complex of aquatic macroinvertebrates has been sampled, with more than 9,000 specimens from 42 taxa collected: Ephemeroptera (*Caenis* Stephens, 1835; *Farrodes* Peters, 1971; *Callibaetis* Eaton, 1881; *Tricorythodes* Ulmer, 1920), Odonata (*Argia* Rambur, 1842; *Homeoura* Kennedy, 1920; *Tramea* Hagen, 1861), Hemiptera (*Belostoma* Latreille, 1807), Coleoptera (*Scirtes* Illiger, 1807; *Berosus* Leach, 1817; *Desmopachria* Babington, 1841; *Hexacylloepus* Hinton 1940a, 1940b; *Hydrochus* Leach, 1817; *Microcylloepus* Hinton 1935); Trichoptera (*Polycentropus* Curtis, 1835; *Oxyethira* Eaton, 1873); Lepidoptera (*Parapoynx* Hübner, 1825) and Diptera (*Dasyhelea* Kieffer, 1911; *Palpomyia* Meigen, 1818; *Atrichopogon* Kieffer, 1906; Chironominae and Tanypodinae). Some of these genera may include new taxa as happened with the Elmidae after a thorough taxonomic study. The ecological and evolutionary significance of Lagoa Misteriosa is particularly relevant in the context of its isolated karstic setting, where hydrological connectivity, habitat persistence, and spatial isolation may collectively influence patterns of aquatic diversification. The relative isolation of karstic water bodies can constrain dispersal and gene flow, potentially promoting genetic differentiation and lineage persistence over evolutionary timescales. In this context, the discovery of two undescribed Elmidae species provides important biogeographical evidence that isolated karst ecosystems may function as localized reservoirs of aquatic biodiversity and potential centers of endemism. The occurrence of distinct elmid lineages in Lagoa Misteriosa further suggests that the biodiversity of such systems may be underestimated, particularly when ecological surveys are predominantly focused on connected lotic environments. Consequently, isolated karst habitats warrant increased attention as potentially important units for understanding patterns of species distribution, diversification, and endemism in Neotropical aquatic beetles

During the study, based on six samples over one year, only two species of two genera of Elmidae were collected; no other elmid taxa have been recorded from Lagoa Misteriosa. Because of this, the larvae were easily associated with the adults, and also the females with the males, since all that was sampled together in association with the rooted macrophyte *Heteranthera zosterifolia*. In the case of *Hexacylloepus misteriosa* **sp. nov.**, both sexes are rather similar, and in *Microcylloepus dimorphus* **sp. nov.,** the high sexual dimorphism was the key for the match, once this was the only *Microcylloepus* specimen collected during one year, and all the males and females presented the same pattern that was described in *Neoelmis guarani*.

For both genera, we are providing the second immature-adult associations and descriptions of the larvae of the species. Until now, the only *Microcylloepus* larva previously described was that of *Microcylloepus inaequalis* from Mexico by Hinton (1940a, 1940b), and for *Hexacylloepus*, only *Hexacylloepus ferrugineus* from the USA had been described by Brown (1973). Unfortunately, Elmidae larval studies are still scant in the Neotropical region; in recent years, just a few publications have focused on the description of immatures (Polizei and Casari 2019; Martinez-Roman et al. 2019; González-Córdoba et al. 2020; Laššová et al. 2023). On the other hand, larvae are often collected in much larger numbers than adults. In this yearlong study, 192 adults and 2,391 larvae of *Microcylloepus* were sampled. Without an accurate association and identification, this material can be useless for some projects. The newly established associations between immature and adult forms of Elmidae provide a valuable basis for future ecological surveys and biomonitoring studies, particularly by facilitating the identification of larval material collected independently of adults. By linking immature stages to their corresponding adult taxa, these associations can enhance the taxonomic resolution of larval-based assessments and broaden the ecological applicability of existing datasets. Consequently, the present contribution extends beyond the taxonomic characterization of the species, providing information that may support a wider range of ecological and biomonitoring applications without the need for additional analyses.

These new species represent the first Brazilian Elmidae described from a lentic habitat. The family is fairly common in streams and rivers (lotic habitats) of tropical and subtropical regions (Jäch and Balke 2008), but not in lentic habitats. Lentic environments are closed systems, such as lakes, ponds, swamps, defined as standing water without continuous flow in a definite direction, besides the absence of riffles or its slow velocity. Their main characteristics, in contrast to lotic environments, are lower oxygen concentrations, less habitat heterogeneity, and less uniform distribution of dissolved or suspended nutrients. These conditions are problematic for species belonging to the large subfamily Elminae, since they have plastron-type respiration and are benthic organisms that live associated with a substrate. This substrate is usually covered by algae and bryophytes which are used as food by these scraper organisms (Elliot 2008). For these reasons, Elmidae species are rarely present in lentic habitats. However, the occurrence of these new species in a lentic environment suggests that such habitats may represent suitable and potentially underexplored habitats for Elmidae than previously recognized 

The greatest number of specimens was collected between October and February, corresponding to the phytoplankton bloom period, when dissolved oxygen concentrations and pH were higher (see Table 1), rendering Lagoa Misteriosa a more suitable habitat for Elmidae riffle beetles. During this period, 104 out of 189 adults and 1,985 out of 2,391 larvae of *Microcylloepus dimorphus* **sp. nov.** were sampled, as well as 7 out of 10 adults of *Hexacylloepus misteriosa* **sp. nov.**. In addition to the elevated dissolved oxygen levels, the reduced water visibility during this period may provide a refuge for these beetles, as the darker water likely decreases predation pressure from fish and other aquatic insects, such as dragonflies.

Until now just a few riffle beetle genera have reported from karst spring or karst lotic habitats: *Anommatelmis* Spangler 1981 and *Lemalelmis* Spangler 1981 from Haiti, *Elmis* Latreille 1802 from Croatia and Slovenia, *Limnius* Illiger 1802 from Marocco and *Typhloelmis* Barr, 2015 from USA (Hernando et al. 2001; Mori and Brancelj 2006; Barr et al. 2015; Stanković et al. 2015; Simović et al. 2024). However, none has been reported from a lentic karst environment.

Concerning the Neotropical region, Taylor and Ferreira (2012) reported two undetermined Elmidae species from a limestone cave in Minas Gerais State, however the cave has a stream that flows downstream from a lake in the epigean system near the entrance into the cave. Thus, these two species may have been swept away and carried by the stream current into the cave. Here we reported the second species of *Microcylloepus* associated with this atypical lentic habitat. The first record was *Microcylloepus similis* Horn 1870 from Montezuma Well, Arizona, USA (Blinn & Sanderson 1989).

Regarding the *Hexacylloepus*, this study represents the first record of the genus in this type of environment. Martins and Ferreira (2020) just reported an undetermined *Hexacylloepus* from a stream, which flows from a cave, in two stretches external (epigean) and subterranean (hypogean) in Montes Claros, Minas Gerais state, Brazil. So, both new species were found in a most unusual situation: in association with the rooted macrophyte *Heteranthera zosterifolia* along banks of Lagoa Misteriosa, in a Neotropical karst sinkhole. This is a remarkable example of how Elmidae studies in lentic Neotropical habitats can be a novel source of beetle diversity.

## Data Availability

Not applicable.
